# Synthesis of a Series of Heterocyclic N‐Substituted Derivatives of Alendronic Acid and Their Preliminary Biological Evaluation

**DOI:** 10.1002/cbdv.71865

**Published:** 2026-10-22

**Authors:** Anna Nasulewicz‐Goldeman, Tomasz M. Goszczyński, Joanna Wietrzyk, Waldemar Goldeman

**Affiliations:** ^1^ Department of Experimental Oncology Hirszfeld Institute of Immunology and Experimental Therapy Polish Academy of Sciences Wrocław Poland; ^2^ Department of Organic and Medicinal Chemistry Faculty of Chemistry Wrocław University of Science and Technology Wrocław Poland

**Keywords:** alendronate, antiproliferative activity, bisphosphonates, bisphosphonic acids, hydroxyapatite

## Abstract

The aim of this work was to examine the influence of introduction of heteroaromatic motifs like pyridyl and imidazole rings into alendronic acid molecule on its biological properties. For this purpose, a series of heterocyclic N‐substituted alendronate derivatives were synthesized via a two‐step, one‐pot procedure. This procedure involves the condensation of alendronate and heteroaromatic aldehydes in the presence of triethylamine, which leads to the formation of appropriate Schiff bases, followed by the in situ reduction of the resulting imines with sodium borohydride. The study investigated the ability of the obtained bisphosphonates to bind to hydroxyapatite (HAP), as well as their antiproliferative effect against RAW 264.7 mouse macrophages, MCF‐7 human breast cancer and MCF‐10A normal breast epithelial cells. Among the tested derivatives, the 2‐imidazole derivative (**4f**) and its *N*‐methylated analog (**4g**) showed the best activity against both RAW 264.7 macrophages and MCF‐7 cells, with an IC_50_ value of 19.4 and 76.1 µM and of 26.4 and 107.9 µM, respectively. Furthermore, all synthesized derivatives were non‐cytotoxic toward MCF‐10A normal cells under the tested experimental conditions. The 4(5)‐imidazole (**4e**) derivative showed the highest affinity to HAP.

## Graphical abstract

The influence of pyridyl and imidazole substituents on the biological properties of alendronate derivatives is investigated. A series of heterocyclic N‐substituted bisphosphonates is synthesized and evaluated for hydroxyapatite binding and antiproliferative activity. Imidazole derivatives exhibit the highest biological activity, while one derivative shows the strongest hydroxyapatite affinity. All compounds remain non‐cytotoxic toward normal cells.

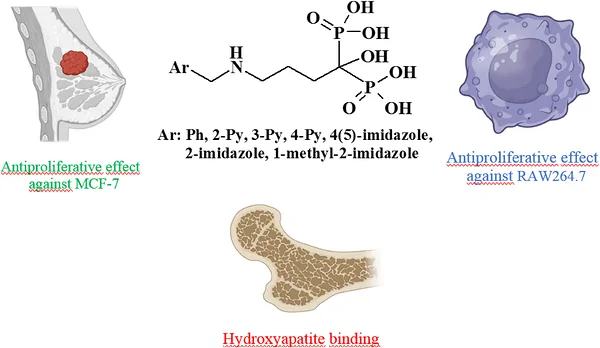

## 1 Introduction

Bisphosphonates (BPs) are a family of compounds containing a methylene bisphosphonic moiety (H_2_O_3_P─C─PO_3_H_2_) which makes them stable analogs of inorganic pyrophosphate. Due to the presence of the bisphosphonic backbone in their structure, BPs show a high affinity to calcium ions and can bind to bone minerals [1, 2, 3]. BPs are the main drugs used in the treatment of Paget's disease [4, 5], and in the treatment and prevention of postmenopausal osteoporosis [6, 7] and other bone metabolism‐related diseases [8]. Bisphosphonic acids are also used to treat hypercalcemia of malignancy [9, 10], tumor‐induced hypercalcemia [11] as well as they exhibit direct and indirect antitumor activity [12, 13]. The most active nitrogen‐containing compounds (*N*‐BPs) belong to the so‐called second‐generation (e.g., alendronate, pamidronate, and ibandronate) and third‐generation (risedronate, minodronate, and zoledronate) BPs (Figure 1).

**FIGURE 1:**
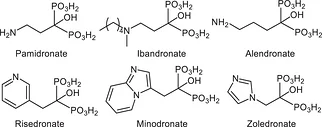
Structures of selected second‐ and third‐generation BPs.

The high affinity of bisphosphonic backbone to hydroxyapatite (HAP) present in bone makes BPs an excellent molecular hook in drug–BP conjugates applied in bone imaging and bone targeting therapies [14, 15]. Due to the presence of the free amino group in their structure, pamidronate and alendronate are widely used in the preparation of drug–BP conjugates. For example, ^68^Ga‐complexes with DOTA‐alendronate [16] or DOTA‐pamidronate [17] conjugates were used as positron emission tomography radiotracers in bone imaging. Polymeric vesicles based on poly‐*ε*‐caprolactone‐poly‐glutamic acid copolymers modified with alendronic acid were found to have potential in single photon emission computed tomography/computed tomography (SPECT/CT) imaging and in drug delivery to malignant bone tumors [18]. Graphene oxide–collagen hydrogels modified with alendronate demonstrated strong antiosteoclastogenic ability [19]. Furthermore, alendronic acid was used in the preparation of a variety of diagnostic probe conjugates such as fluorescein‐ [20], indocyanine green‐ [21], naphthalimide‐ [22], and BODIPY‐alendronate [23].

Alendronate is a first‐choice drug in the treatment of osteoporosis and hypercalcemia [24]. It has been shown to reduce bone turnover, improve bone mineral density [25, 26], and exhibit antifracture effect in postmenopausal osteoporotic women [27, 28]. That makes it the most commonly used BPs in the treatment of osteoporosis. It is known that the antiresorptive activity of BPs and their affinity to HAP depend on the nature of the substituent attached to the central carbon atom of the BP moiety (H_2_O_3_P─C─PO_3_H_2_) [29]. However, in spite of a great biological potential of alendronic acid, only few examples of its simple N‐substituted derivatives (like *N*‐methyl, *N*‐ethyl, *N*‐iso‐butyl, and *N*‐pentyl) are described in the literature. Most of the derivatives of alendronate are conjugates of various drugs and alendronate via amide bond. Thus, the possibility of a simple modification of terminal free amino group of alendronate as well as its strong activity prompted us to prepare a series of simple nitrogen‐modified alendronate derivatives as potential antiresorptive and anticancer BPs in this study.

It is well established that second‐generation BPs containing a nitrogen atom in an aliphatic side chain are considerably more active than the simple first‐generation BPs, such as etidronate and clodronate. Even greater activity has been observed for derivatives in which the nitrogen atom is incorporated into a heterocyclic ring, as in third‐generation BPs. The presence and nature of the nitrogen atom in the side chain not only influence the biological activity and mechanism of action of *N*‐BPs but also affect their affinity to HAP by enabling the formation of additional hydrogen bonds involving the nitrogen atom [30]. Therefore, it appears that the introduction of a heterocyclic moiety into the side chain of a bisphosphonate may enhance its biological activity. This hypothesis is consistent with the findings of Savino et al., who have shown that introducing the pyrimidine ring into the 2‐amino‐1‐hydroxyethanebisphosphonic acid (H_2_NCH_2_C(OH)(PO_3_H_2_)_2_) significantly increased its activity toward human tumor prostate cancer PC3 cells and RAW 264.7 murine macrophages [31]. Therefore, we decided to modify the structure of alendronate by introducing additional nitrogen atom(s) into heterocyclic moieties, such as pyridine and imidazole rings, which are present in the structures of risedronate and zoledronate. We then examined the influence of these structural modifications on the antiproliferative activity and affinity to HAP of the obtained BPs.

## 2 Results and Discussion

The majority of strategies used for the synthesis of N‐substituted alendronate derivatives are based on the formation of an amide bond [32, 33, 34, 35, 36]. In this study, we aimed to develop a simple methodology for the preparation of these derivatives, while maintaining the basic character of the nitrogen atom and simultaneously introducing pyridine or imidazole ring into the structure of alendronate. Therefore, we modified and applied a procedure described for the preparation of *N*‐(3‐thiophenemethyl)alendronate, which was used as a ligand for polyoxometalate complexes [37]. The title bisphosphonic acids were prepared directly from alendronic acid (**2**) via a two‐step, one‐pot procedure. In the first step of the procedure, aromatic aldehydes (**1a–g**) were condensed with alendronate in the presence of triethylamine as a base, which led to the formation of corresponding Schiff bases (**3a–g**). In the next step, the resulting imines (**3a–g**) were subjected to in situ reduction with sodium borohydride (Scheme 1) and neutralized with hydrochloric acid. Neutralization caused the precipitation of crude products, which were recrystallized from the appropriate solvent to obtain desired bisphosphonic acids (**4a–g**).

**SCHEME 1:**
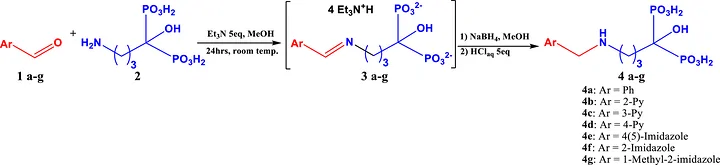
General scheme for the synthesis of N‐substituted alendronate derivatives (**4a**–**g**).

Using the above‐described procedure, a series of 2‐, 3‐, and 4‐pyridyl, as well as 2‐ and 4(5)‐imidazole derivatives of alendronate, were obtained. To evaluate the influence of heteroaromatic motifs on the biological activity and binding of these derivatives to HAP, a simple *N*‐benzylated derivative was also prepared (**4a**). Finally, seven N‐substituted alendronate derivatives (**4a**–**g**) were prepared. The structures of all the derivatives were confirmed by ^1^H, ^13^C, and ^31^P NMR (Figures S8–S31), ATR‐FTIR (Figures S32–S39), and ESI–MS. The introduction of the arylmethyl (ArCH_2_‐) moiety, resulting from the reduction of the imine group, was confirmed by the presence of characteristic signals of methylene protons at 3.6–3.7 ppm in the ^1^H NMR spectra, along with the corresponding signals assigned to the protons of the appropriate aromatic group. Both the integration and the multiplicity of the individual ^1^H NMR signals unequivocally confirm the presence of the respective aromatic moiety. The presence of an additional ‐CH_2_‐ carbon signal at approx. 50 ppm in the ^13^C NMR spectra, together with the characteristic signals of the corresponding aromatic group in the 120–160 ppm region, also clearly demonstrates the presence of the respective arylmethyl moieties in the molecules. The ATR‐FTIR spectra of the synthesized products are highly complex. Nevertheless, they provide evidence for the presence of phosphonate groups as well as the aromatic moieties introduced into the alendronate scaffold. The spectra display characteristic absorption bands in the regions of approximately 800–900, 1000–1100, and 1150–1200 cm^−^^1^, which can be assigned to vibrations involving P─C, P─O, and P═O bonds, respectively. Bands associated with the C═C and C═N stretching vibrations of the unsaturated aromatic and heteroaromatic moieties are observed in the region around 1600 cm^−^^1^. The spectra also exhibit absorption bands attributable to O─H stretching vibrations, typically appearing as broad bands above 3200 cm^−^^1^, as well as C─H stretching vibrations in the 3000–3150 cm^−^^1^ region. In addition, broad absorption features observed at approximately 2000–2200 cm^−^^1^ may be associated with strongly hydrogen‐bonded O─H groups of the phosphonic acid moieties [38, 39, 40].

At this point it is worth mentioning, that, in contrast to the classical methods based on phosphonylation of carboxylic acids, presented approach allows a preparation of the new BPs by simple, direct modification of alendronate.

Bisphosphonic acids are known to exhibit a high affinity to calcium HAP [1, 2, 3, 41, 42, 43]. In the case of 1‐hydroxybisphosphonic acids, the affinity is increased by the presence of hydroxyl group at the geminal carbon atom because chelation occurs by tridentate binding [1, 44]. To evaluate the affinity to HAP, all the synthesized BPs were incubated with HAP at blood pH (Tris‐buffer, 50 mM, pH 7.4) according to a slightly modified literature procedure [3, 42]. After 5, 30, and 120 min, and 24 h of incubation, the percentage of BPs left in the solution was determined by UV–vis spectroscopy (Figures S1–S7). The obtained results are shown in Table 1 and Figure S40. Due to the lack of any absorption bands above 195 nm in the alendronate spectrum, we were not able to compare its affinity to HAP with the affinity of the studied compounds.

**TABLE 1:** Hydroxyapatite (HAP) binding of the studied BPs, expressed as the percentage of a bound compound.

Compound nos.	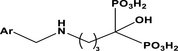	Affinity to HAP^a^ [%]^b^			
		5 min	30 min	2h	24h
**4a**	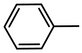	35.9 ± 1.9	39.8 ± 1.7	42.6 ± 2.4	49.6 ± 0.8
**4b**	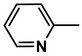	35.2 ± 3.7	39.7 ± 3.3	42.5 ± 4.2	50.6 ± 1.8
**4c**	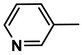	40.0 ± 1.2	45.1 ± 0.8	49.5 ± 3.0	56.9 ± 2.2
**4d**	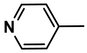	39.7 ± 1.0	44.0 ± 0.8	48.7 ± 4.8	54.7 ± 0.6
**4e**	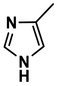	46.1 ± 0.9	50.8 ± 0.5	54.0 ± 1.8	59.5 ± 0.4
**4f**	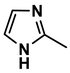	34.7 ± 0.7	38.3 ± 0.8	41.5 ± 0.5	46.2 ± 0.5
**4g**	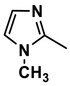	40.1 ± 0.3	44.6 ± 0.7	47.7 ± 1.0	53.8 ± 2.4

^a^Data presented as the mean and standard deviation of three experiments.

^b^Percentage of the BP bound to HAP.

As expected, a time‐dependent decrease in absorbance at the selected wavelength was observed in all cases, indicating the HAP‐binding capability of the investigated BPs. The concentration of the studied BPs in solution decreased substantially within the first 5 min of incubation. Among the investigated compounds, the 4(5)‐imidazole derivative **4e** exhibited the highest affinity for HAP. Approximately 46% of **4e** was bound to HAP after 5 min, increasing to 60% after 24 h of incubation. The affinity of **4e** for HAP was significantly higher than that of all other derivatives at all time points (*p* < 0.001 for **4a**, **4b**, **4d**, and **4f**, and *p* < 0.01 for **4c** and **4g**), with the exception of compound **4c** after 24 h, for which the difference was not statistically significant. It is noteworthy that the constitutional isomer of **4e**, the 2‐imidazole derivative **4f**, exhibited a significantly lower affinity for HAP than derivatives **4c**–**4g**. The differences were most pronounced after 24 h of incubation, when the average percentage bound to HAP was 46% for **4f**, compared with 57% for **4c** (*p* < 0.0001), 55% for **4d** (*p* < 0.0001), 60% for **4e** (*p* < 0.0001), and 54% for **4g** (*p* < 0.001). The higher affinity of **4g** compared with **4f** indicates that *N* ^1^‐methylation of the 2‐imidazole ring increased its affinity for HAP. No significant differences in HAP affinity were observed among compounds **4a**, **4b**, and **4f**, with approximately 35% and 42% bound after 5 min and 2 h of incubation, respectively. After 24 h, bisphosphonate **4f** exhibited a slightly lower affinity for HAP than **4a** and **4b** (46% vs. 50%, *p* > 0.05).

The bone microenvironment is a supportive niche for cancer cell survival and tumor growth. BPs display a high affinity to bone tissue and strongly influence this environment. In this way, they not only diminish the resorptive processes mediated by osteoclasts but also inhibit the development of tumor metastatic foci in bones. Therefore, we evaluated the antiproliferative activity of the synthesized BPs against MCF‐7 breast adenocarcinoma cells as a model of cancer which preferentially metastasizes to bone. In addition, we analyzed the activity of the tested BPs against RAW 264.7 macrophages, a model of osteoclasts. Murine macrophages are considered as convenient osteoclast surrogates for in vitro studies characterizing the pharmacological properties of BPs since these cells are derived from the same hematopoietic lineage, highly endocytic, and capable of demineralizing bone particles [45].

The results of the antiproliferative activity analysis of the N‐substituted alendronate derivatives (**4**) are summarized in Table 2 and in Figure S41.

**TABLE 2:** Antiproliferative activity of the studied BPs (**4a‐g**) and the control compounds against RAW 264.7 murine macrophages, MCF‐7 human breast adenocarcinoma, and MCF10A human normal epithelium cells.

Compound nos.	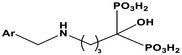	IC_50_ [µM]^a^			cLogP^c^	TPSA^c^ [Å^2^]
		RAW 264.7	MCF‐7	MCF10A		
**4a**	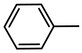	32.6 ± 2.1	358.6 ± 111.0	UN^b^	−1.28	178.26
**4b**	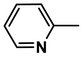	43.6 ± 17.6	287.6 ± 147.2	UN^b^	−2.18	191.18
**4c**	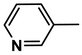	43.8 ± 14.5	UN^b^	UN^b^	−2.22	191.18
**4d**	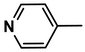	35.7 ± 8.0	UN^b^	UN^b^	−2.19	191.18
**4e**	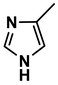	38.3 ± 8.3	350.6 ± 187.5	UN^b^	−2.84	206.94
**4f**	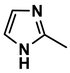	19.4 ± 9.6 [1]	76.1 ± 18.8 [4]	UN^b^	−2.84	206.94
**4g**	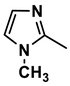	26.4 ± 3.4	107.9 ± 36.9 [4]	UN^b^	−3.23	196.08
*Alendronate*	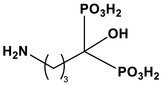	2.5 ± 1.2 [2]	30.5 ± 15.3	277.7 ± 51.9	−3.11	192.25
*Cisplatin*		1.2 ± 0.4 [2]	7.0 ± 2.6 [5]	6.9 ± 2.5	—	—

^a^IC_50_ values were determined at concentrations in the range of 1–1000 µM. Values are mean ± standard deviation.

^b^UN‐not active in the tested concentration range.

^c^Consensus LogP and TPSA (topological polar surface area) values were predicted using the online SwissADME web tool; ^[1]^—significant versus **4b**, **4c**, **4e**, *p* < 0.05; ^[2]^—significant versus **4a‐4g**, *p* < 0.0001; ^[3]^—significant versus **4a**, **4b**, **4e**, p<0.05; ^[4]^—significant versus **4a**, **4e**, *p* < 0.05; ^[5]^—significant versus alendronate, *p* < 0.001.

None of the BPs tested showed antiproliferative activity toward normal MCF10A epithelial cells, whereas cisplatin, used as the reference drug, showed IC_50_ value of approx. 7 µM. The activity of *N*‐benzyl alendronate (**4a**) toward RAW 264.7 macrophages was similar to that of the pyridyl (**4b**–**d**) and 4(5)‐imidazole (**4e**) derivatives, with IC_50_ values ranging from 36 to 45 µM, which is five‐ to seven‐fold higher than that of alendronate. 2‐Imidazole derivatives **4f** and **4g** were found to be the most interesting among the tested compounds as they showed antiproliferative potential against RAW 264.7 macrophages with IC_50_ values of approximately 19 and 26 µM, respectively. Moreover, both these compounds inhibited the proliferation of MCF‐7 human breast adenocarcinoma cells. Interestingly, while the three pyridyl derivatives exhibit similar activity against both the RAW 264.7 and MCF‐7 cell lines and are practically inactive against the latter, the three imidazole derivatives display more diverse activity profiles, with derivatives **4e** and **4f** being active against MCF‐7 cells. It can cautiously be suggested that substitution of the imidazole ring at the 2‐position is more favorable than at the 4(5)‐position. Both the 2‐imidazole derivative **4f** and its *N*‐methyl derivative **4g** are more active than the corresponding 4(5)‐imidazole regioisomer **4e**, an effect that is particularly evident in MCF‐7 cells. Interestingly, among the imidazole derivatives, the less biologically active 4(5)‐substituted derivative **4e** exhibited the highest affinity for HAP. This difference might result from a more favorable spatial orientation of its imidazole nitrogen atoms, potentially facilitating the formation of an additional hydrogen‐bonding interaction with a hydroxyl group of HAP [29]. Analysis of cLogP values does not reveal a straightforward correlation between the hydrophobicity of the BPs and their antiproliferative activity. For example, compound **4a**, the most hydrophobic derivative, exhibits comparable activity against RAW 264.7 and MCF‐7 cells to derivative **4e**, despite a difference of approx. 1.5 cLogP units, corresponding to more than one order of magnitude in lipophilicity. However, analysis of cLogP values indicates that the most active compounds **4f** and **4g** are the most hydrophilic derivatives and exhibit cLogP values closest to that of the control alendronate. Replacement of the phenyl ring with pyridyl or imidazolyl moieties alters the electronic distribution within the molecules owing to the presence of nitrogen atom(s), increasing their polarity and hydrogen‐bonding capability. However, similarly to the analysis of hydrophobicity, no clear relationship between the electronic properties of the substituents and biological activity can be established. Despite differences in the electronic character of the phenyl and pyridyl substituents, their biological activities remain comparable. More pronounced differences are observed only for the imidazole derivatives, where the presence of two nitrogen atoms may more substantially modulate the electron distribution and hydrogen‐bonding capability of the molecules. Table 2 also presents the calculated TPSA values. However, the calculated TPSA values do not reveal any clear relationship with the observed biological activity. Overall, the introduction of an arylmethyl fragment of varying hydrophobicity into the structure of alendronate appears to be associated with a decrease in antiproliferative activity against both RAW 264.7 and MCF‐7 cells. Nevertheless, the antiproliferative activity of the derivatives **4f** and **4g** toward above cell lines, together with their high affinity to HAP, indicated their potential antiresorptive activity and modulatory effects on tumor‐associated macrophages.

## 3 Conclusions

A series of pyridyl‐ and imidazolyl‐containing N‐substituted derivatives of alendronic acid (**4a–g**) was synthesized via a two‐step, one‐pot procedure. This procedure involves the condensation of alendronate and heteroaromatic aldehydes in the presence of triethylamine, which leads to the formation of appropriate Schiff bases, followed by in situ reduction of the resulting imines with sodium borohydride and neutralization with hydrochloric acid. To estimate the influence of above heteroaromatic motifs introduction into alendronic acid molecule on the biological properties, we also evaluated the ability of the obtained BPs to bind to HAP as well as their antiproliferative effect against RAW 264.7 mouse macrophages and MCF‐7 human breast cancer cells. All obtained BPs have shown lower activity toward RAW 264.7 macrophages and MCF‐7 cells than control alendronate, however the imidazole derivatives were the most active. The 2‐imidazole derivative **4f** and its *N*‐methylated analog **4g** showed the best antiproliferative activity against RAW 264.7 macrophages and MCF‐7 cells, with IC_50_ values of 19.4 and 76.1 µM and of 26.4 and 173.1 µM, respectively. Furthermore, all derivatives showed high affinity to HAP. The strongest binding properties were observed in the case of 4(5)‐imidazole **4e** derivative. The present study has several limitations. First, the relatively small number of compounds investigated does not allow for a comprehensive structure–activity relationship (SAR) analysis. Second, due to technical limitations, the binding data obtained for the synthesized compounds can only be compared with one another and cannot be directly related to the reference alendronate.

## 4 Experimental Section

### 4.1 Materials and Methods

^1^H, ^13^C, and ^31^P NMR spectra were recorded on a Bruker Avance 300 (at 300, 75 and 121 MHz, respectively) and Jeol ECZ 400S spectrometers (at 400, 100 and 162 MHz, respectively) in D_2_O/NaOD (Sigma–Aldrich) and locked on deuterium from a solvent. The following abbreviations for multiplicity were used: s = singlet, d = doublet, t = triplet, q = quartet, m = multiplet, and br = broad. High‐resolution mass spectra were recorded on a MicrOTOF‐Q II spectrometer (Bruker Daltonic, Bremen, Germany) equipped with an electrospray ion source. The instrument was operated in the negative‐ion mode and calibrated with a sodium formate solution (10 mM). UV–vis spectra were recorded on J‐1500 spectropolarimeter (Jasco) equipped with thermostated cell holder and PM‐539 detector under nitrogen atmosphere. ATR‐FTIR spectra were collected using a Bruker Vertex 70v Fourier transform infrared spectrophotometer equipped with an air‐cooled DTGS detector and diamond attenuated total reflection infrared cell at 2 cm^−1^ resolution and 64 scans in the middle‐infrared (4000–400 cm^−1^) at room temperature. Melting points were determined using a Electrothermal Mel‐Temp 1102D melting point apparatus and were uncorrected. All solvents were of commercial quality and purchased from a local supplier (Avantor Performance Materials Poland S.A.). Starting aldehydes and other reagents were purchased from Merck (phosphorous trichloride 99%, Cat. no. 157791, CAS no. 7719‐12‐2; 4‐aminobutyric acid >99%, Cat. no. A5835, CAS no. 56‐12‐2; benzaldehyde >99%, Cat. no. B1334, CAS no. 100‐52‐7; 2‐pyridinecarboxaldehyde 99%, Cat. no. P62003, CAS No. 1121‐60‐4; 3‐pyridinecarboxaldehyde 98%, Cat. no. P62208, CAS no. 500‐22‐1; 4‐pyridinecarboxaldehyde 97%, Cat. no. P62402, CAS no. 872‐85‐5; 4‐imidazolecarboxaldehyde 98%, Cat. no. P62402, CAS no.3034‐50‐2; 2‐imidazolecarboxaldehyde 97% Cat. no. 272000, CAS no. 10111‐08‐7; 1‐methyl‐2‐imidazolecarboxaldehyde 98%, Cat. no. 475122, CAS no. 13750‐81‐7; sodium borohydride 99%, Cat. no. 213462, CAS no. 16940‐66‐2; triethylamine >99.5%, Cat. no. 471283, CAS no. 121‐44‐8). No procedures or isolations of products were optimized. Alendronate was prepared using modification of the Rachon's procedure [46]. Consensus logP (cLogP) and TPSA were calculated using the SwissADME web tool (https://www.swissadme.ch). The cLogP values were calculated as the arithmetic mean of four prediction models implemented in SwissADME (XLOGP3, WLOGP, MLOGP, and SILICOS‐IT).

### 4.2 Cell Culture

RAW 264.7 mouse macrophages (RRID: CVCL_0493) and MCF‐7 human breast adenocarcinoma cells (RRID: CVCL_0031) were obtained from European Collection of Authenticated Cell Cultures (Salisbury, UK), and MCF10A human normal epithelium (RRID: CVCL_0598) from American Type Culture Collection (Rockville, Maryland, USA) and maintained in the Cell Culture Collection of the Institute of Immunology and Experimental Therapy (IIET), Polish Academy of Sciences, Wroclaw, Poland. RAW264.7 cells were maintained in DMEM (Gibco, Scotland, UK) supplemented with 2 mM L‐glutamine and 10% fetal bovine serum (FBS; both from Sigma–Aldrich, Darmstadt, Germany). MCF‐7 cells were cultured in Eagle's medium (IIET, Wroclaw, Poland) with 10% FBS, 10 µg mL^−1^ insulin, 10 µg ml^−1^ hydrocortisone, 2 mM L‐glutamine (all from Sigma–Aldrich, Germany) and amino acids (Gibco Thermo Fisher Scientific Inc., UK). MCF10A cells were cultured in Ham's F‐12 medium (Corning, Arizona, USA) with 7.5% horse serum (Gibco Thermo Fisher Scientific Inc., UK), 10 µg ml^−1^ insulin, 10 µg ml^−1^ hydrocortisone, 0.5 µg ml^−1^ cholera toxin, 2 mM L‐glutamine (Sigma–Aldrich, Germany) and amino acids (Gibco Thermo Fisher Scientific Inc., UK). All culture media were supplemented with 100 U mL^−1^ of penicillin (Polfa Tarchomin, Poland) and 0.1 mg mL^−1^ of streptomycin (Sigma–Aldrich, Germany). Cell lines were cultured in humid atmosphere at 37°C and 5% CO2.

### 4.3 Experimental Procedures

#### 4.3.1 Preparation of Alendronic Acid (**2**)

A solution of phosphorous trichloride (104.0 mL, 1.2 mol) was vigorously stirred magnetically. To this, a solution of 4‐aminobutyric acid (20.6 g, 0.20 mol) in a mixture of water (14 mL) and 12 M hydrochloric acid (17 mL) was added dropwise at 0°C (water–ice bath) for 1 h. Then, the reaction mixture was gradually heated to about 80°C (water bath temperature) under a reflux condenser connected to hydrochloride absorber. After about 1.5 h of heating, a thick semisolid phase was obtained which could not be stirred further. The two‐phase reaction mixture was heated additionally for about 30 min at the same temperature and subsequently cooled to room temperature. Excess PCl_3_ was removed by decantation, and the remaining was removed along with other volatile material by evaporation. The resulting residue was dissolved in water (150 mL) and refluxed for about 4 h, after which about 5 g of activated charcoal was added. After about 2 h of further heating, the charcoal was separated by suction and washed with hot water (3 × 30 mL). The collected filtrates were evaporated under vacuum, the residue was dissolved in water (100 mL), and the solution was evaporated again. The dissolution–evaporation procedure was repeated five times to obtain a semisolid residue, which was treated with water (60 mL) and stirred for about 30 min. The resulting solid was washed with water (3 × 20 mL) and then with methanol (3 × 20 mL), and finally dried in air to obtain alendronic acid monohydrate.

#### 4.3.2 General Procedure for the Preparation of N‐Substituted Alendronic Acid Derivatives (**4a–g**)

To a suspension of alendronic acid monohydrate (2.7 g, 10 mmol) in methanol (50 mL), a portion of triethylamine (7.0 mL, 50 mmol) was added, and the mixture was magnetically stirred for about 30 min to obtain a milky solution. Then, the aldehyde (10.5 mmol) was added dropwise at room temperature for 10 min, and the reaction mixture was stirred for 24 h. The resulting clear solution was cooled to about 0°C (water–ice bath), and NaBH_4_ (1.9 g, 50 mmol) was added carefully in small portions. Next, the reaction mixture was gradually heated to room temperature and stirred for 48 h. The solution was subsequently cooled to about 0°C and cautiously acidified with 12 M aqueous hydrochloric acid (∼8.3 mL). The resulting suspension was stirred for about 1 h at 0°C. The precipitate was filtered, washed with methanol (4 × 10 mL), and dissolved in hot water (100 mL). About 3 g of activated charcoal was added, and the mixture was refluxed for 2 h. Then, the charcoal was separated by suction and washed with hot water (3 × 20 mL). The collected filtrates were evaporated under vacuum, and the crude product was crystallized, as described for individual BPs.

#### 4.3.3 Binding of **4a–g** to Hydroxyapatite (HAP)

HAP (100,0 mg, CH ceramic hydroxyapatite, Type II, 20 µm, BIO‐RAD Cat. No.158‐2200) in Tris‐buffer (5 mL, 50 mM, pH 7.4) was stirred on rotary shaker (30 rpm) for 1 h at room temperature. 2 mM solutions of **4a**–**g** were (5 mL, the same buffer) added to the above HAP suspension. The resulting mixture was stirred on rotary shaker (30 rpm). After 5 min, 30 min, 2 h, and 24 h aliquots of 1 mL were centrifuged (20 k × g), a 200 µL sample of supernatant was taken and concentrations of the studied BPs were determined by UV spectroscopy at *λ* = 205,8 (**4a**); 201,6 (**4b**); 200,8 (**4c**); 259 (**4d**); 201 (**4e** and **4f**), 220 (**4g**) nm.

#### 4.3.4 Antiproliferative Assays

Cells were plated in 96‐well plates (Sarstedt, Inc. Newton, NC, USA) at a density of 1 × 10^4^ cells per well in 100 µL of culture medium without FBS and antibiotics. After 24 h of incubation under standard conditions, cells were treated with BPs (at serial concentrations of 1000 – 100 – 10 – 1 µM, each concentration in triplicates). An SRB assay was performed after additional 72 h as described by Skehan et al. [47]. The optical densities of the samples were measured by a Synergy H4 Hybrid Reader (BioTek Instruments, Winooski, VT, USA) at *λ* = 540 nm. Mean proliferation inhibition was calculated for each concentration of the compound based on crude absorbance utilizing formula: % Inh = 100 – ((Mean Abs X—Mean Abs M)/(Mean Abs CTRL—Mean Abs M)) × 100, where Mean Abs X—mean absorbance in compound‐treated wells; Mean Abs CTRL—mean absorbance in nontreated wells; Mean Abs M—mean absorbance in cell‐free wells. The IC_50_ are absolute values were calculated based on the inhibition of proliferation rates using Prolab software (INFORM‐TECH, Zabrze, Poland) adapted from Cheburator 0.4, Dmitry Nevozhay software (version 1.2.0 software by Dmitry Nevozhay, 2004–2014, http://www.cheburator.nevozhay.com/, freely available, [48]) for each experiment. Each experiment was repeated at least 3 times independently. IC_50_ values were log‐transformed and subsequently assessed for compliance with the assumptions of normality. Statistical analysis was performed using one‐way ANOVA followed by Tukey's post hoc test. For the MCF10A cell line, an unpaired test was used.

#### 4.3.5 Alendronate (**2**)

White crystalline solid; yield: 22.5 g (44%). ^1^H NMR (400 MHz, D_2_O+NaOD): *δ* = 1.42–1.52 (m, 2H, CH_2_), 1.54–1.67 (m, 2H, CH_2_), 2.33 (t, *J* = 7.2 Hz, 2H, CH_2_); ^13^C{^1^H} NMR (100 MHz, D_2_O+NaOD): *δ* = 27.41 (t, *J* = 4.5 Hz), 33.41, 41.64, 76.35 (t, *J* = 140.0 Hz);^31^P{^1^H} NMR (162 MHz, D_2_O+NaOD): *δ* = 19.62. ATR‐FTIR: 461, 522, 564, 663, 724, 750, 825, 921 (P─C), 930, 964, 987, 1054 (P─O), 1108 (P─O), 1162, 1204 (P═O), 1315, 1373, 1471, 1497, 1605 (NH^+^), 1628 (NH^+^), 1672, 2051 (PO─H), 2162 (PO─H), 2917 (C─H), 3090, 3144 cm^−1^. All spectra (Figures S8–S10) are in good agreement with literature data [49, 50]. For copies of the NMR and ATR‐FTIR spectra, see Figures S8–S10 and S32, respectively.

#### 4.3.6 4‐(Benzylamino)‐1‐hydroxybutylidene Diphosphonic Acid (**4a**)

White solid (crystallized three times from boiling water); m.p. 239°C–242°C (dec); yield: 0.7 g (20%). ^1^H NMR (300 MHz, D_2_O+NaOD): *δ* = 1.56–1.73 (m, 4H, 2 × CH_2_), 2.38 (t, *J* = 6.9 Hz, 2H, CH_2_), 3.55 (s, 2H, PhCH_2_), 7.13–7.26 (m, 5H, ArH). ^13^C{^1^H} NMR (75 MHz, D_2_O+NaOD): *δ* = 22.40, 32.28, 48.21, 51.39, 74.83 (t, *J* = 130.3 Hz), 128.52, 128.85, 129.23, 133.89. ^31^P{^1^H} NMR (121 MHz, D_2_O+NaOD): *δ* = 19.08 (s). HRMS‐ESI(‐): *m*/*z* [M–H]^−^ calcd for C_11_H_19_NO_7_P_2_: 338.0553; found: 338.0570. ATR‐FTIR: 455, 501, 555, 596, 672, 698, 736, 770, 873, 897 (P─C), 927, 972, 1041 (P─O), 1077, 1097 (P─O), 1148, 1200 (P═O), 1234 (P═O), 1331, 1380, 1406, 1444, 1496, 1621 (C═C), 2323 (PO─H), 2833, 2950 (C─H), 2977 (C─H), 3031 (C─H) cm^−1^. For copies of the NMR and ATR‐FTIR spectra, see Figures S11–S13 and S33, respectively.

#### 4.3.7 4‐(2‐Pyridinylmethylamino)‐1‐hydroxybutylidene Diphosphonic Acid (**4b**)

White solid (crystallized twice from water/methanol mixture 1:1, *v*/*v*); m.p. 193°C–198°C (dec); yield: 1.7 g (50%). ^1^H NMR (400 MHz, D_2_O+NaOD): *δ* = 1.61–1.76 (m, 4H, 2 × CH_2_), 2.41 (t, *J* = 6.8 Hz, 2H, CH_2_), 3.69 (s, 2H, PyCH_2_), 7.18 (dd, *J* = 5.6, 7.2 Hz, 1H, PyH), 7.29 (d, *J* = 8.0 Hz, 1H, PyH), 7.68 (dt, *J* = 2.0, 8.0 Hz, 1H, PyH), 8.29 (br d, *J* = 4.8 Hz, 1H, PyH). ^13^C{^1^H} NMR (75 MHz, D_2_O+NaOD): *δ* = 24.29 (t, *J* = 7.0 Hz), 33.66, 49.42, 53.49, 76.25 (t, *J* = 133.5 Hz), 122.69, 123.05, 138.04, 148.19, 158.31. ^31^P{^1^H} NMR (162 MHz, D_2_O+NaOD): *δ* = 19.46 (s). HRMS‐ESI(‐): *m*/*z* [M–H]^−^ calcd for C_10_H_18_N_2_O_7_P_2_: 339.0506; found: 339.0521. ATR‐FTIR: 439, 508, 568, 602, 625, 666, 768, 913 (P─C), 1054 (P─O), 1125 (P═O), 1445, 1472, 1549, 1596 (C═C, C═N), 1623 (C═C, C═N), 2100 (PO─H), 2323 (PO─H), 2659, 2957 (C─H) cm^−1^. For copies of the NMR and ATR‐FTIR spectra, see Figures S14–S16 and S34, respectively.

#### 4.3.8 4‐(3‐Pyridinylmethylamino)‐1‐hydroxybutylidene Diphosphonic Acid (**4c**)

White solid (crystallized once from water at *ca*. 5°C); m.p. 228°C–232°C (dec); yield: 2.7 g (79%). ^1^H NMR (300 MHz, D_2_O+NaOD): *δ* = 1.65–1.82 (m, 4H, 2 × CH_2_), 2.47 (t, *J* = 6.2 Hz, 2H, CH_2_), 3.67 (s, 2H, PyCH_2_), 7.32 (dd, *J* = 5.0 Hz, *J* = 8.0 Hz, 1H, PyH), 7.73 (d, *J* = 8.0 Hz, 1H, PyH), 8.32 (d, *J* = 4.5 Hz, 1H, PyH), 8.37 (s, 1H, PyH). ^13^C{^1^H} NMR (75 MHz, D_2_O+NaOD): *δ* = 23.13, 31.97 (br s), 48.61, 49.14, 74.37 (bt, *J* = 127.7 Hz), 124.19, 132.64 (bs), 138.18, 147.97, 148.85. ^31^P{^1^H} NMR (121 MHz, D_2_O+NaOD): *δ* = 18.94 (s). HRMS‐ESI(‐): *m*/*z* [M–H]^−^ calcd for C_10_H_18_N_2_O_7_P_2_: 339.0506; found: 339.0519. ATR‐FTIR: 433, 437, 514, 546, 612, 628, 681, 781, 867, 890 (P─C), 912 (P─C), 986, 1053 (P─O), 1143 (P═O), 1275, 1382, 1438, 1475, 1497, 1599 (C═C, C═N), 1638 (C═C, C═N), 1980, 2169 (PO─H), 3069 (C─H), 3300 (O─H) cm^−1^. For copies of the NMR and ATR‐FTIR spectra, see Figures S17–S19 and S35, respectively.

#### 4.3.9 4‐(4‐Pyridinylmethylamino)‐1‐hydroxybutylidene Diphosphonic Acid (**4d**)

White solid (crystallized once from water at *ca*. 5°C); m.p. 255°C–258°C (dec); yield: 2.8 g (81%). ^1^H NMR (300 MHz, D_2_O+NaOD): δ = 1.66–1.78 (m, 4H, 2 × CH_2_), 2.41 (t, *J* = 6.2 Hz, 2H, CH_2_), 3.63 (s, 2H, PyCH_2_), 7.23 (d, *J* = 5.1 Hz, 2H, PyH), 8.29 (d, *J* = 5.1 Hz, 2H, PyH). ^13^C{^1^H} NMR (100 MHz, D_2_O+NaOD): *δ* = 24.24 (t, *J* = 4.8 Hz), 33.71, 49.49, 51.44, 76.26 (t, *J* = 134.2 Hz), 123.76, 148.59, 149.97. ^31^P{^1^H} NMR (121 MHz, D_2_O+NaOD): *δ* = 19.03 (s). HRMS‐ESI(‐): *m*/*z* [M–H]^−^ calcd for C_10_H_18_N_2_O_7_P_2_: 339.0506; found: 339.0524. ATR‐FTIR: 456, 513, 602, 678, 807, 878, 921 (P─C), 978 (P─O), 1055 (P─O), 1140 (P═O), 1271, 1304, 1385, 1473, 1509, 1630 (C═C, C═N), 1645 (C═C, C═N), 2050, 2163 (PO─H), 3103 (C─H), 3235 (O─H) cm^−1^. For copies of the NMR and ATR‐FTIR spectra, see Figures S20–S22 and S36, respectively.

#### 4.3.10 4‐[(1*H*‐Imidazol‐4‐yl)methylamino]‐1‐hydroxybutylidene Diphosphonic Acid (**4e**)

White solid (crystallized twice from water/methanol mixture 1:4, *v*/*v* and next once from water at *ca*. 5°C); m.p. 251°C–253°C (dec); yield: 1.9 g (58%). ^1^H NMR (400 MHz, D_2_O+NaOD): *δ* = 1.58‐1.76 (m, 4H, 2 × CH_2_), 2.40 (t, *J* = 5.2 Hz, 2H, CH_2_), 3.56 (s, 2H, imidazoleCH_2_), 6.85 (s, 1H, imidazoleH), 7.50 (s, 1H, imidazoleH). ^13^C{^1^H} NMR (100 MHz, D_2_O+NaOD): *δ* = 22.69 (t, *J* = 6.0 Hz), 32.14, 43.75, 48.09, 74.66 (t, *J* = 132.9 Hz), 118.27, 132.18, 136.51. ^31^P{^1^H} NMR (162 MHz, D_2_O+NaOD): *δ* = 19.42 (s). HRMS‐ESI(‐): m/z [M–H]^−^ calcd for C_8_H_17_N_3_O_7_P_2_: 328.0458; found: 328.0471. ATR‐FTIR: 446, 476, 488, 517, 538, 611, 626, 729, 745, 849, 877, 901 (P─C), 967 (P─O), 1014, 1057, 1091, 1145 (P═O), 1265, 1304, 1390, 1435, 1475, 1491, 1628 (C═C, C═N), 1665 (C═C, C═N), 1935, 1980 (PO─H), 2014, 2170, 2475 (NH), 2590 (NH), 2716, 3008 (C─H), 3150 (C─H), 3223 (O─H) cm^−1^. For copies of the NMR and ATR‐FTIR spectra, see Figures S23–S25 and S37, respectively.

#### 4.3.11 4‐[(1*H*‐Imidazol‐2‐yl)methylamino]‐1‐hydroxybutylidene Diphosphonic Acid (**4f**)

White solid (crystallized from water/acetone mixture (3:1, *v*/*v*) and next from water); m.p. >260°C (dec); yield: 2.1 g (64%). ^1^H NMR (300 MHz, D_2_O+NaOD): *δ* = 1.5–1.8 (m, 4H, 2 × CH_2_), 2.42 (t, *J* = 6.4 Hz, 2H, CH_2_), 3.56 (s, 2H, imidazoleCH_2_), 6.88 (s, 1H, imidazoleH), 7.48 (s, 1H, imidazoleH). ^13^C{^1^H} NMR (75 MHz, D_2_O+NaOD): *δ* = 23.7, 33.40, 43.98, 48.79, 75.96 (t, *J* = 133.4 Hz), 117.52, 134.52, 136.00. ^31^P{^1^H} NMR (121 MHz, D_2_O+NaOD): *δ* = 18.94 (s). HRMS‐ESI(‐): *m/z* [M‐H]^−^ calcd for C_8_H_17_N_3_O_7_P_2_: 328.0458; found: 328.0468. ATR‐FTIR: 413, 436, 461, 479, 510, 526, 536, 577, 639, 666, 746, 833, 873, 889 (P─C), 919, 955, 961, 981, 1019 (P─O), 1033 (P─O), 1050 (P─O), 1069, 1131 (P═O), 1266, 1277, 1300, 1366, 1434, 1468, 1497, 1595 (C═C, C═N), 1627 (C═C, C═N), 1728, 1942, 2402 (N─H), 2446, 2540, 2568 (N─H), 2632, 2709, 2741, 2925, 2964, 3106 (C─H), 3144 (C─H) cm^−1^. For copies of the NMR and ATR‐FTIR spectra, see Figures S26–S28 and S38, respectively.

#### 4.3.12 4‐[(1‐Methyl‐1*H*‐imidazol‐2‐yl)methylamino]‐1‐hydroxybutylidene Diphosphonic Acid (**4g**)

White solid (crystallized from water/acetone mixture 1:3, *v*/*v* and next from water/EtOH mixture 1:3, *v*/*v*); m.p. > 260°C (dec); yield: 2.8 g (81%). ^1^H NMR (400 MHz, D_2_O+NaOD): *δ* = 1.61‐1.78 (m, 4H, 2 × CH_2_), 2.43 (t, *J* = 7.0 Hz, 2H, CH_2_), 3.52 (s, 3H, CH_3_), 3.67 (s, 2H, imidazoleCH_2_), 6.77 (s, 1H, imidazoleH), 6.90 (s, 1H, imidazoleH). ^13^C{^1^H} NMR (150 MHz, D_2_O+NaOD): *δ* = 23.96 (t, *J* = 6.1 Hz), 32.24, 32.46, 42.99, 49.24, 74.60 (t, *J* = 128.8 Hz), 122.33, 125.83, 146.56. ^31^P{^1^H} NMR (162 MHz, D_2_O+NaOD): *δ* = 19.45 (s). HRMS‐ESI(‐): *m*/*z* [M‐H]^−^ calcd for C_9_H_19_N_3_O_7_P_2_: 342.0614; found: 342.0637. ATR‐FTIR: 414, 450, 518, 528, 644, 683, 729, 747, 875, 906 (P─C), 979 (P─O), 1055, 1162 (P═O), 1281, 1465, 1496, 1532, 1616 (C═C, C═N), 1661 (C═C, C═N), 1981, 2168, 2324 (N─H), 2851, 2923 (C─H), 2959 (C─H), 3300 (O─H) cm^−1^. For copies of the NMR and ATR‐FTIR spectra, see Figures S29–S31 and S39, respectively.

## Author Contributions

**Anna Nasulewicz‐Goldeman**: conceptualization, investigation, formal analysis, writing – review and editing, and writing – original draft. **Tomasz M. Goszczyński**: investigation, writing – review and editing, and writing – original draft. **Joanna Wietrzyk**: funding acquisition and resources. **Waldemar Goldeman**: funding acquisition, conceptualization, investigation, formal analysis, writing – review and editing, and writing – original draft.

## Conflicts of Interest

The authors declare no conflicts of interest.

## Supporting information



Supporting Information for this article (exemplary UV–vis absorption spectral changes of the studied compounds observed upon binding to HAP (Figures. S1–S7), ^1^H, ^31^P{^1^H}, and ^13^C{^1^H}NMR spectra (Figures S8–S31), ATR FT‐IR spectra (Figures S32–S39), time‐dependent binding curves of the studied BPs to HAP (Figure S40) and dose–response curves for all BPs (Figure S41) is available on the WWW under https://doi.org/10.1002/MS‐number.
**Supporting File 1**: cbdv71865‐sup‐0001‐SuppMat.pdf.

## Data Availability

The data that supports the findings of this study are available in the Supporting Information of this study.
