# Supplementary material for: Synthesis of a Series of Heterocyclic N‐Substituted Derivatives of Alendronic Acid and Their Preliminary Biological Evaluation
Source: Chem Biodivers. 2026 Oct 22;23(10):e71865. doi: 10.1002/cbdv.71865 (PMC13687914; doi:10.1002/cbdv.71865)
Supplement: Supplementary file 1 [file CBDV-23-e71865-s001.pdf]

Supplementary Material

Synthesis of a series of heterocyclic *N*-substituted derivatives of alendronic acid and their preliminary biological evaluation

Anna Nasulewicz-Goldeman,[b] Tomasz, M. Goszczyński,[b] Joanna Wietrzyk,[b] Waldemar Goldeman,[*a]

[a] Department of Organic and Medicinal Chemistry, Faculty of Chemistry, Wrocław University of Science and Technology, Wybrzeze Wyspianskiego 27, 50-370 Wrocław, Poland, e-mail: waldemar.goldeman@pwr.edu.pl

[b] Department of Experimental Oncology, Hirszfeld Institute of Immunology and Experimental Therapy, Polish Academy of Sciences, Weigla 12, 53-114 Wrocław

Table of contents

Exemplary UV-Vis absorption spectral changes of the studied compounds (**4a-g**) observed upon binding to HAP in Tris buffer.	Fig. S1-S7
^{1}H, $^{31}P\{^{1}H\}$ and $^{13}C\{^{1}H\}$NMR spectra of all BPs.	Fig. S8-S31
ATR FT-IR spectra of all BPs.	Fig. S32-S39
Time-dependent binding curves of the studied BPs to hydroxyapatite (HAP).	Fig. S40
Dose–response curves for all BPs.	Fig. S41

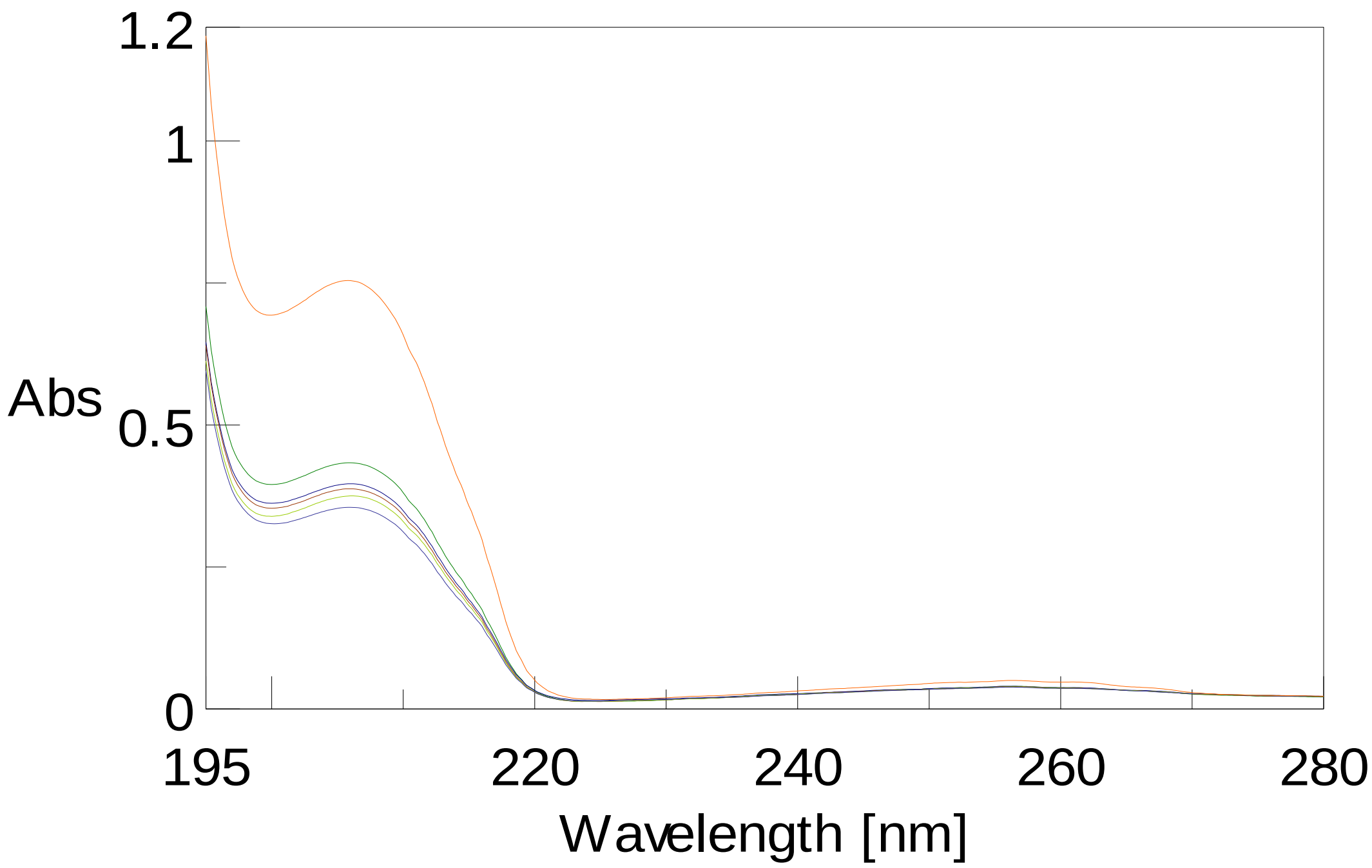

Figure S1. Exemplary UV-Vis absorption spectral changes of **4a** observed upon binding to HAP in Tris buffer.

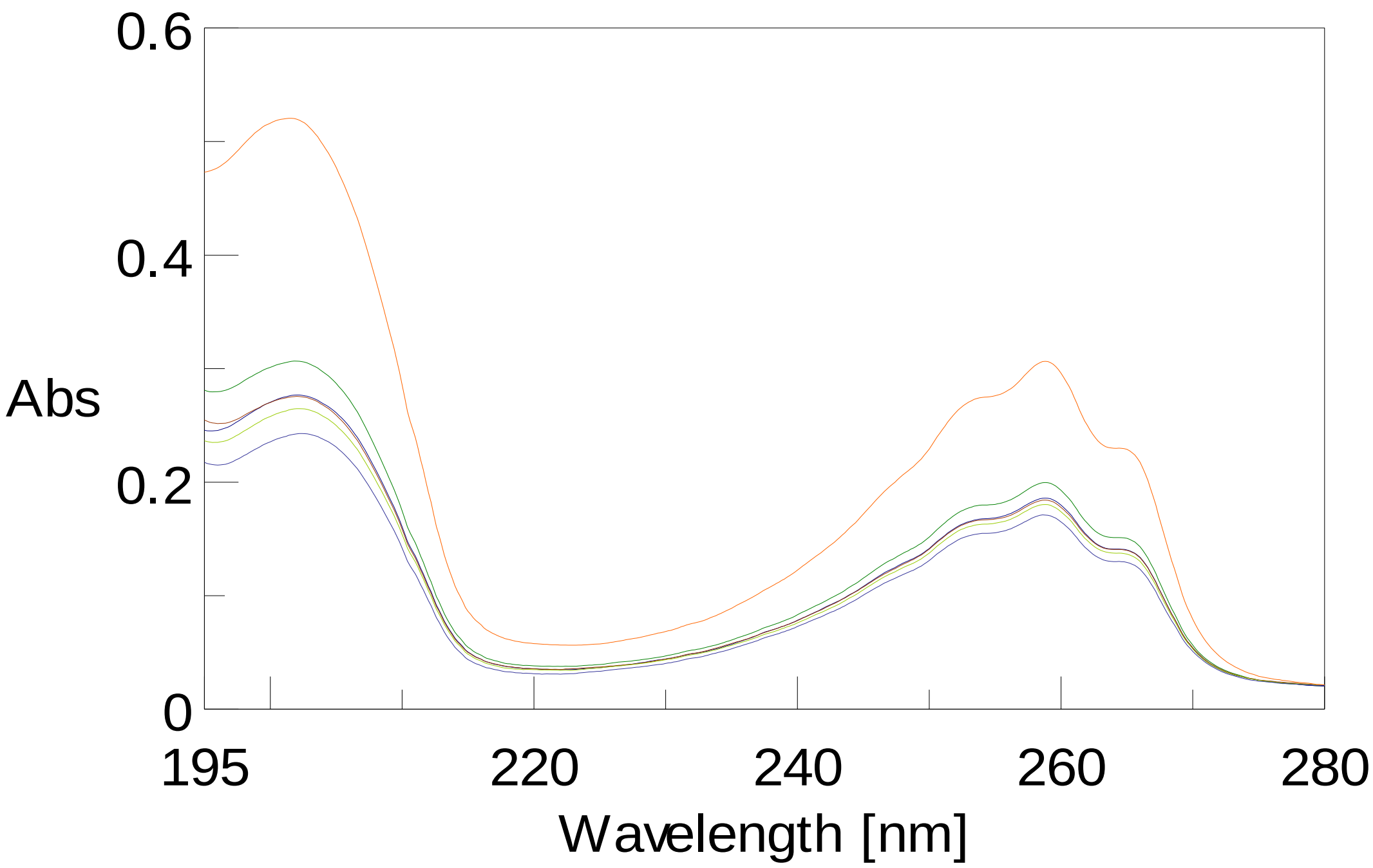

Figure S2. Exemplary UV-Vis absorption spectral changes of **4b** observed upon binding to HAP in Tris buffer.

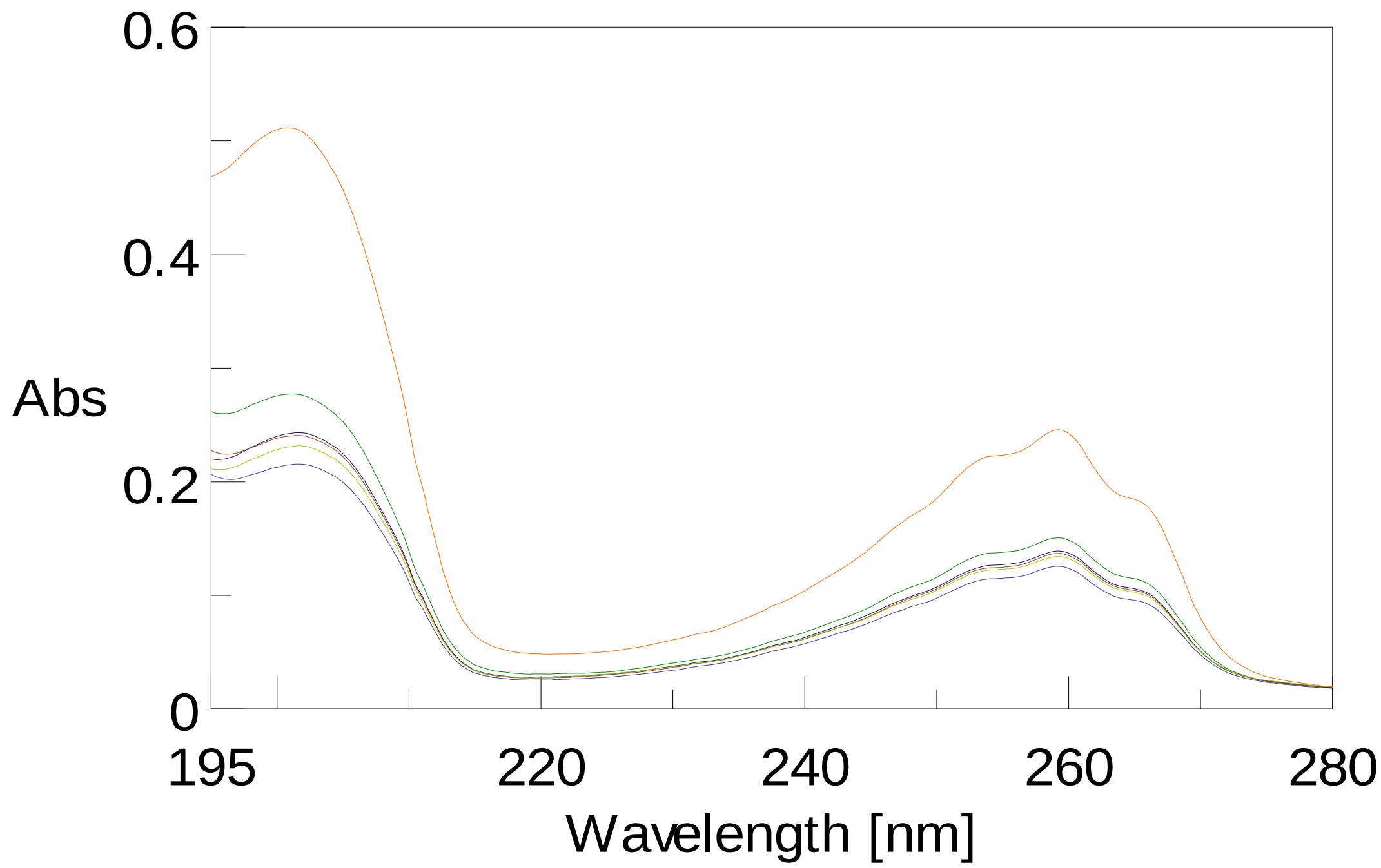

Figure S3.Exemplary UV-Vis absorption spectral changes of **4c** observed upon binding to HAP in Tris buffer.

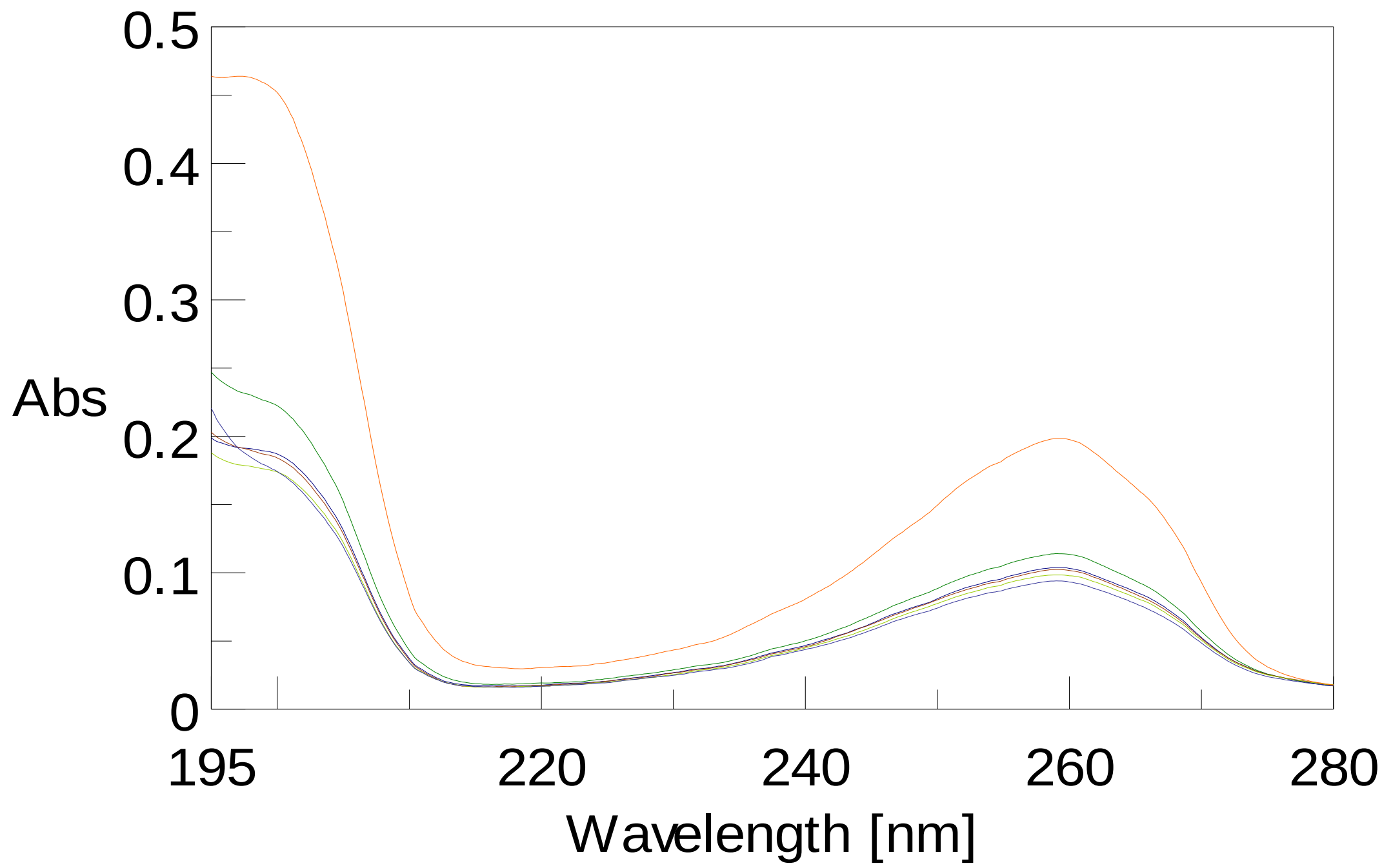

Figure S4. Exemplary UV-Vis absorption spectral changes of **4d** observed upon binding to HAP in Tris buffer.

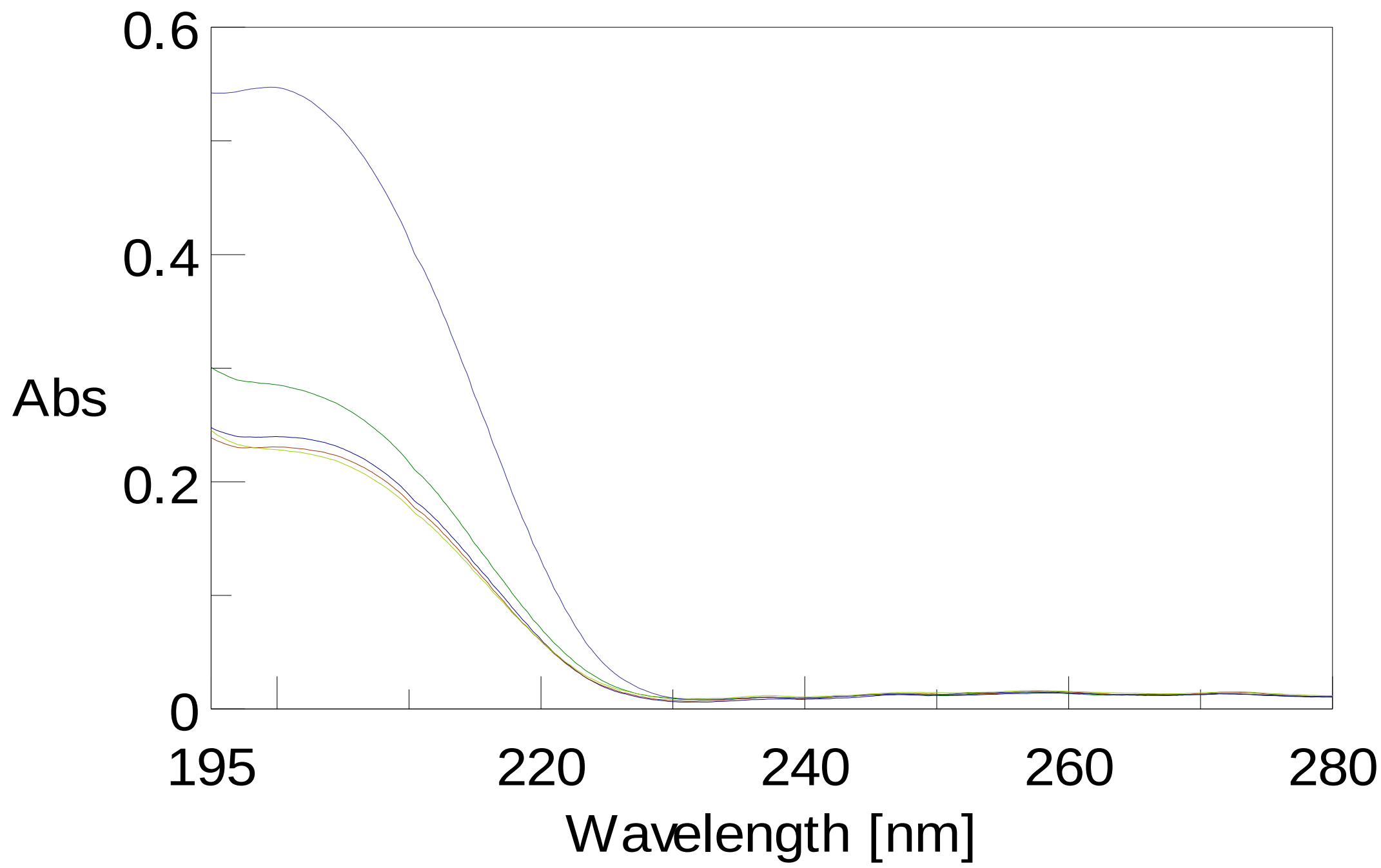

Figure S5. Exemplary UV-Vis absorption spectral changes of **4e** observed upon binding to HAP in Tris buffer.

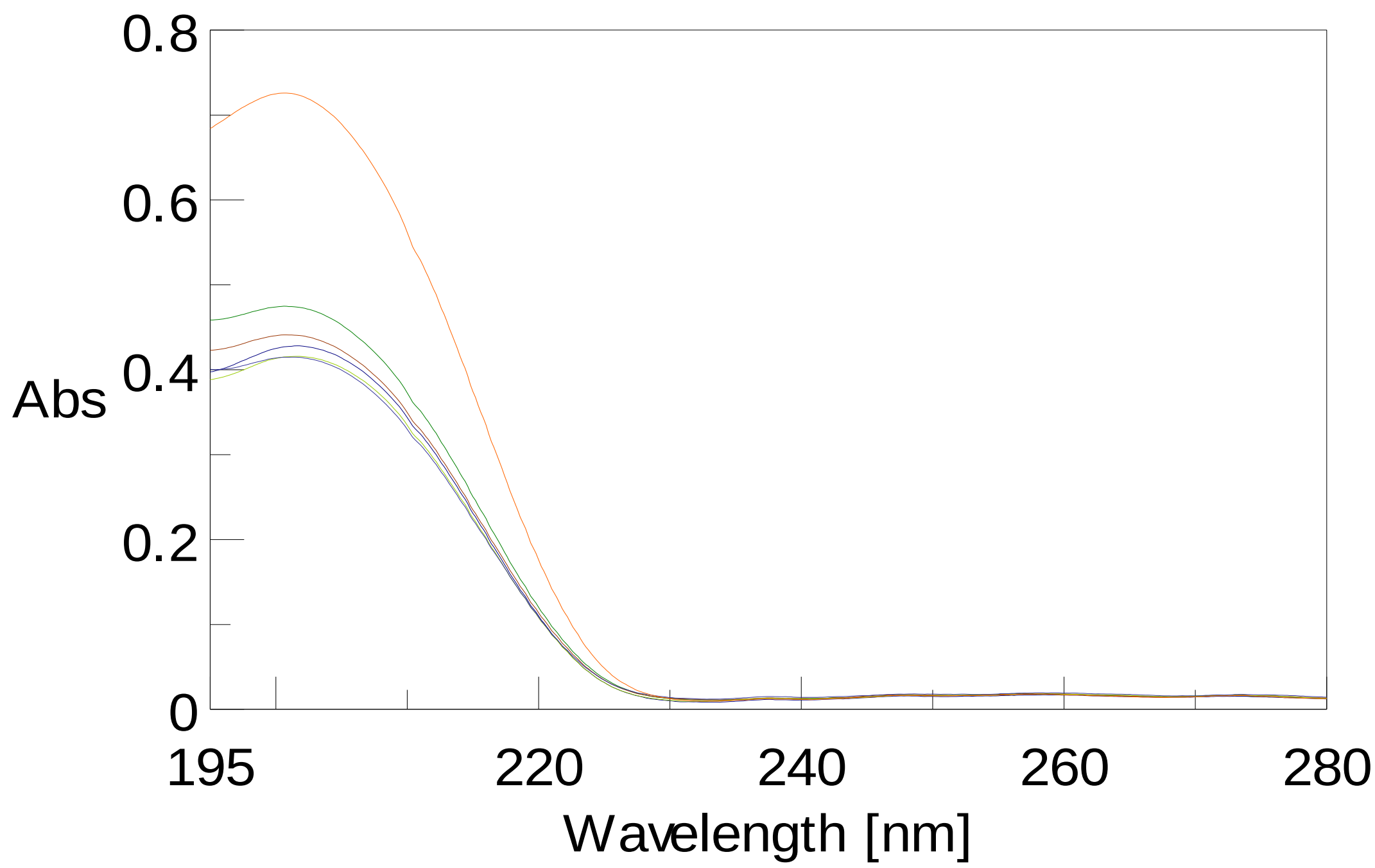

Figure S6. Exemplary UV-Vis absorption spectral changes of **4f** observed upon binding to HAP in Tris buffer.

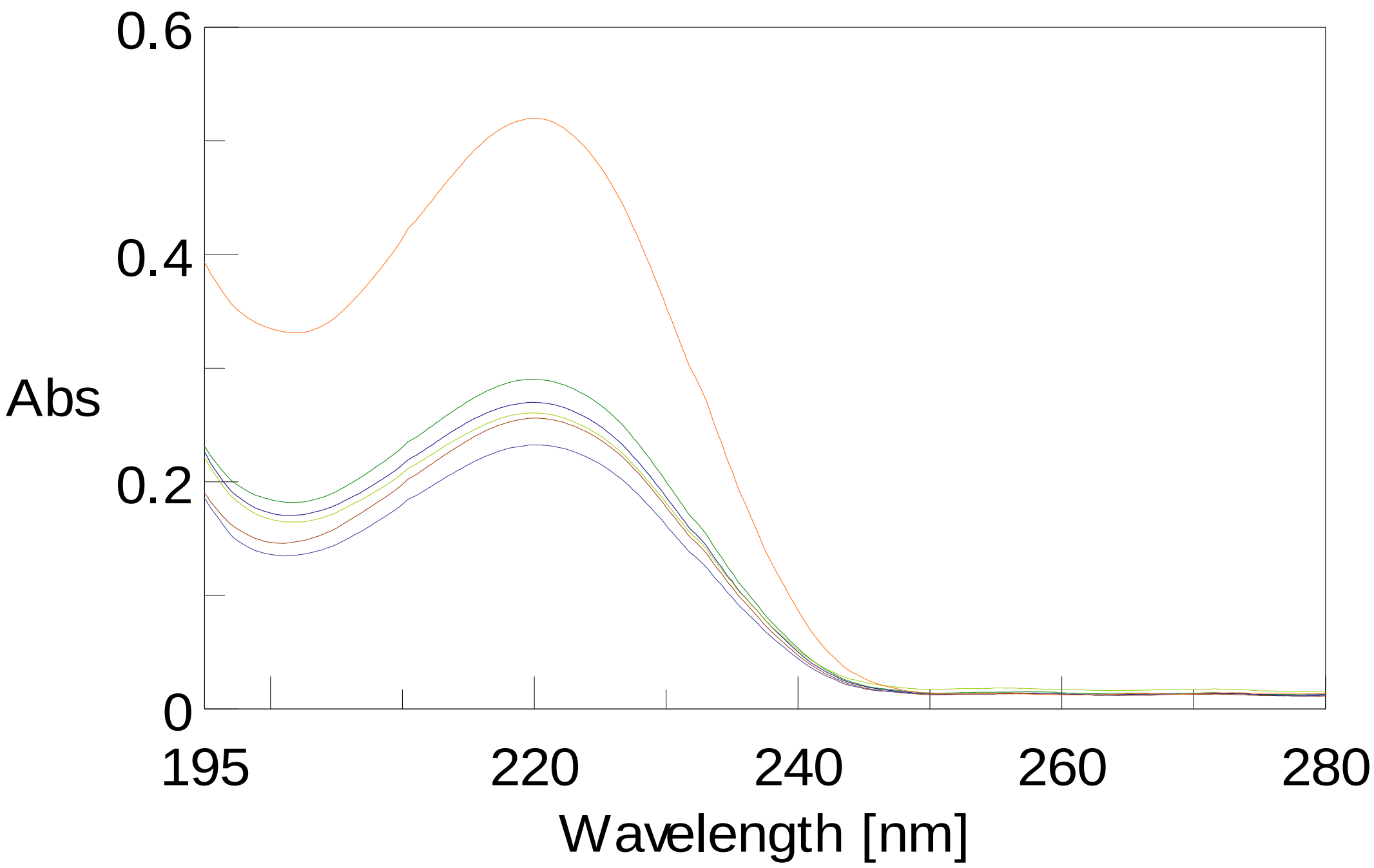

Figure S7. Exemplary UV-Vis absorption spectral changes of **4g** observed upon binding to HAP in Tris buffer.

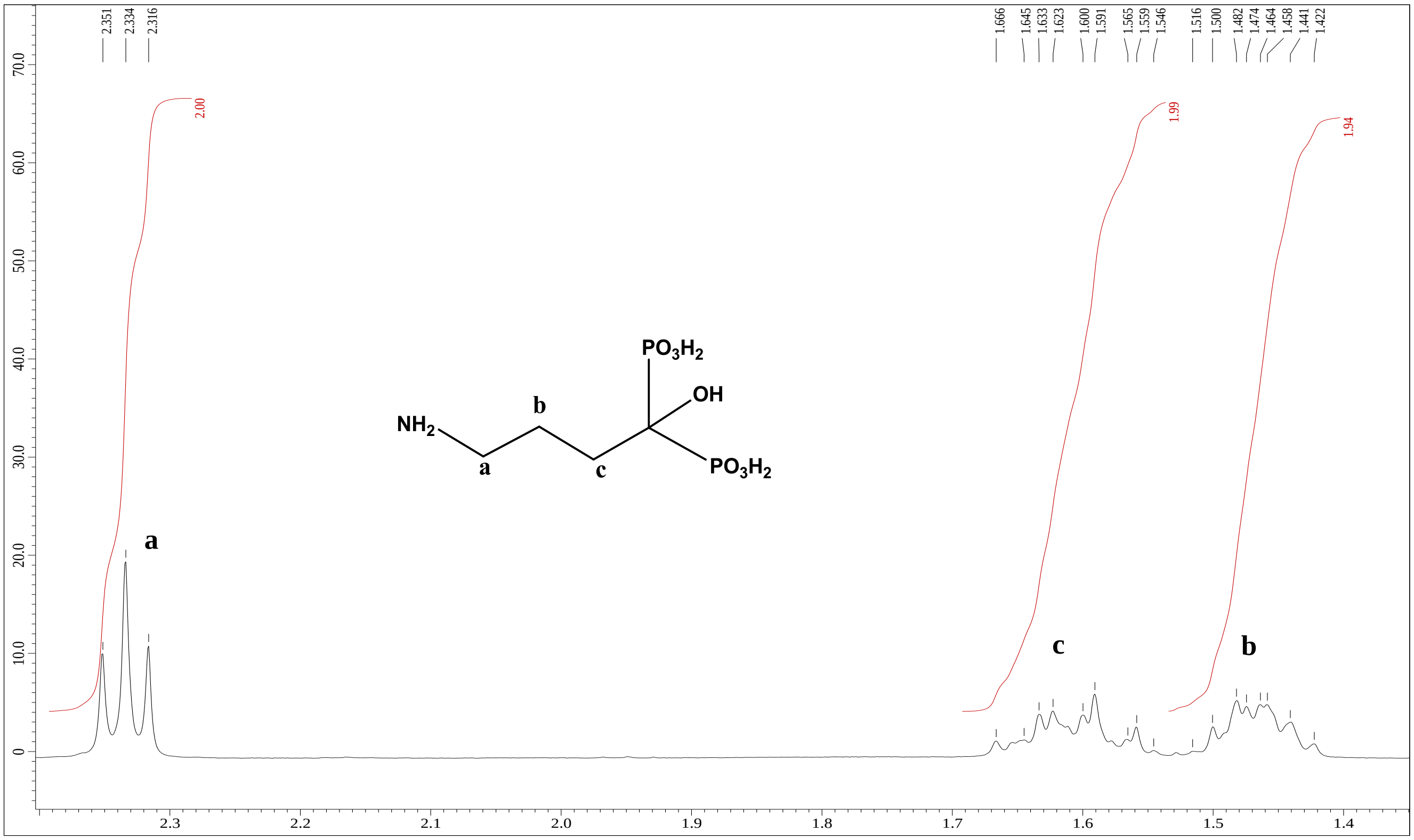

Figure S8. ^1H NMR (400 MHz) spectrum of alendronic acid monohydrate (**2**) in D_2O/NaOD**.**

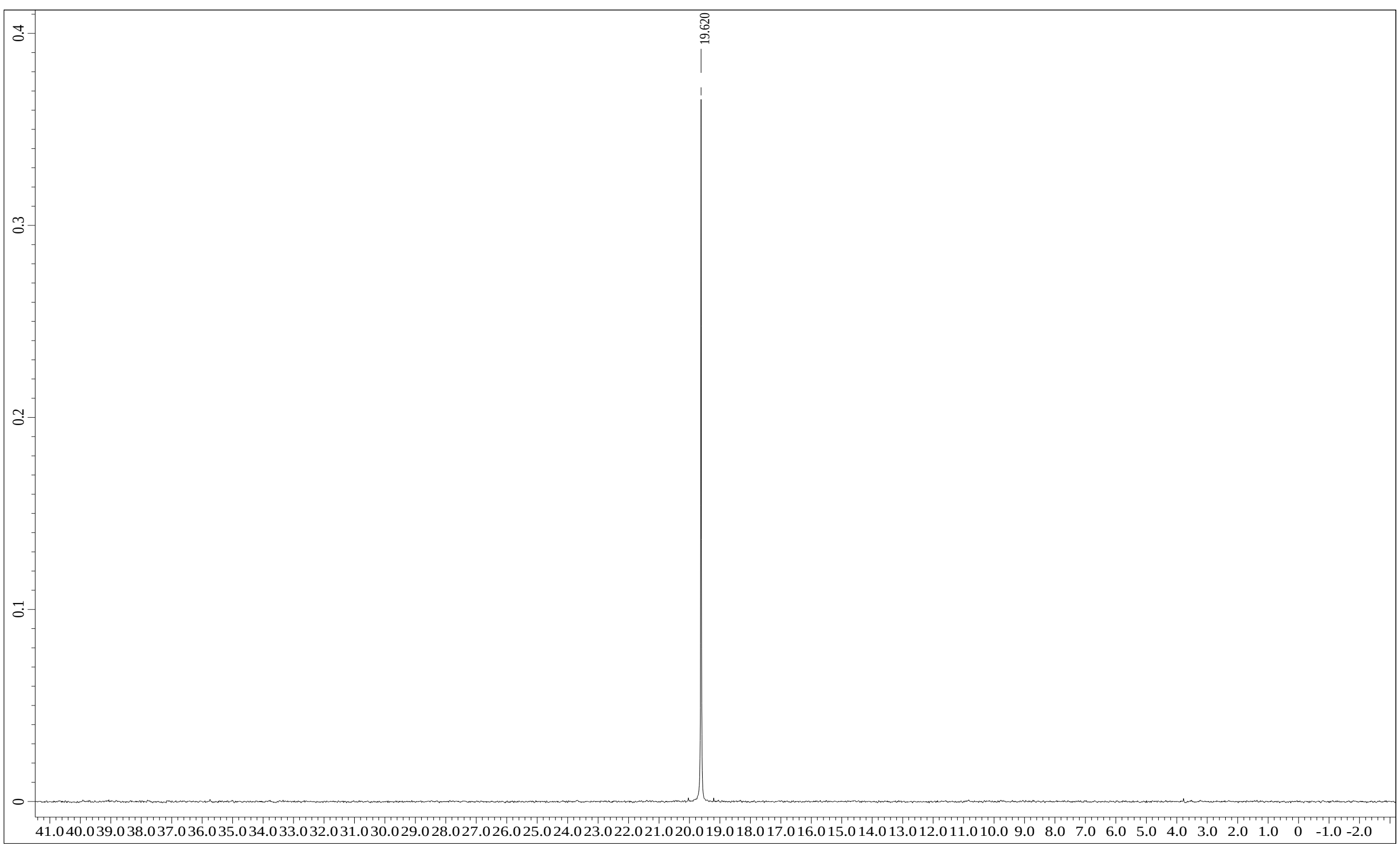

Figure S9. $^{31}P\{^1H\}$ NMR (162 MHz) spectrum of alendronic acid monohydrate (**2**) in D_2O/NaOD.

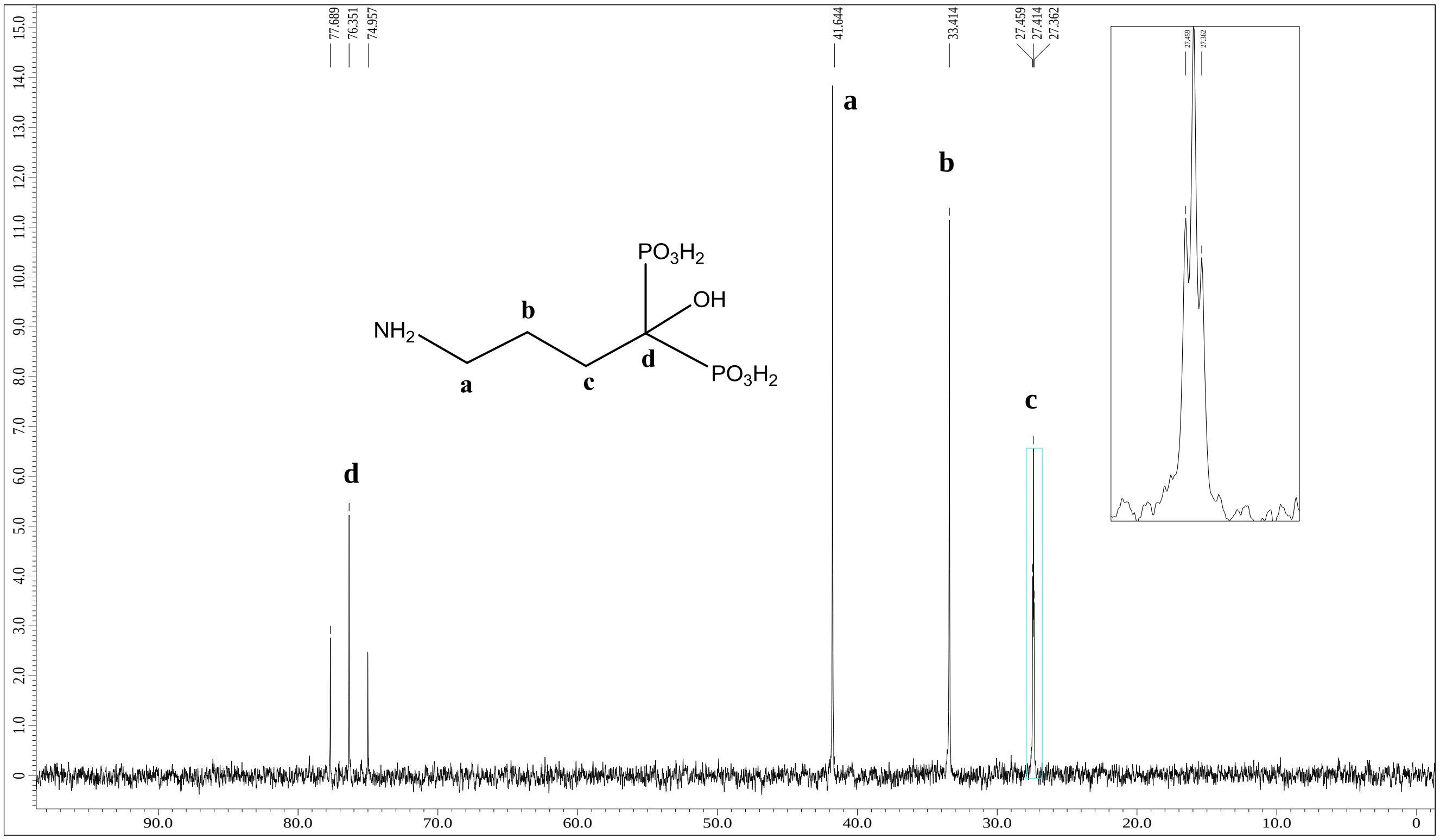

Figure S10. $^{13}C\{^1H\}$ NMR (100 MHz) spectrum of alendronic acid monohydrate (**2**) in D_2O/NaOD.

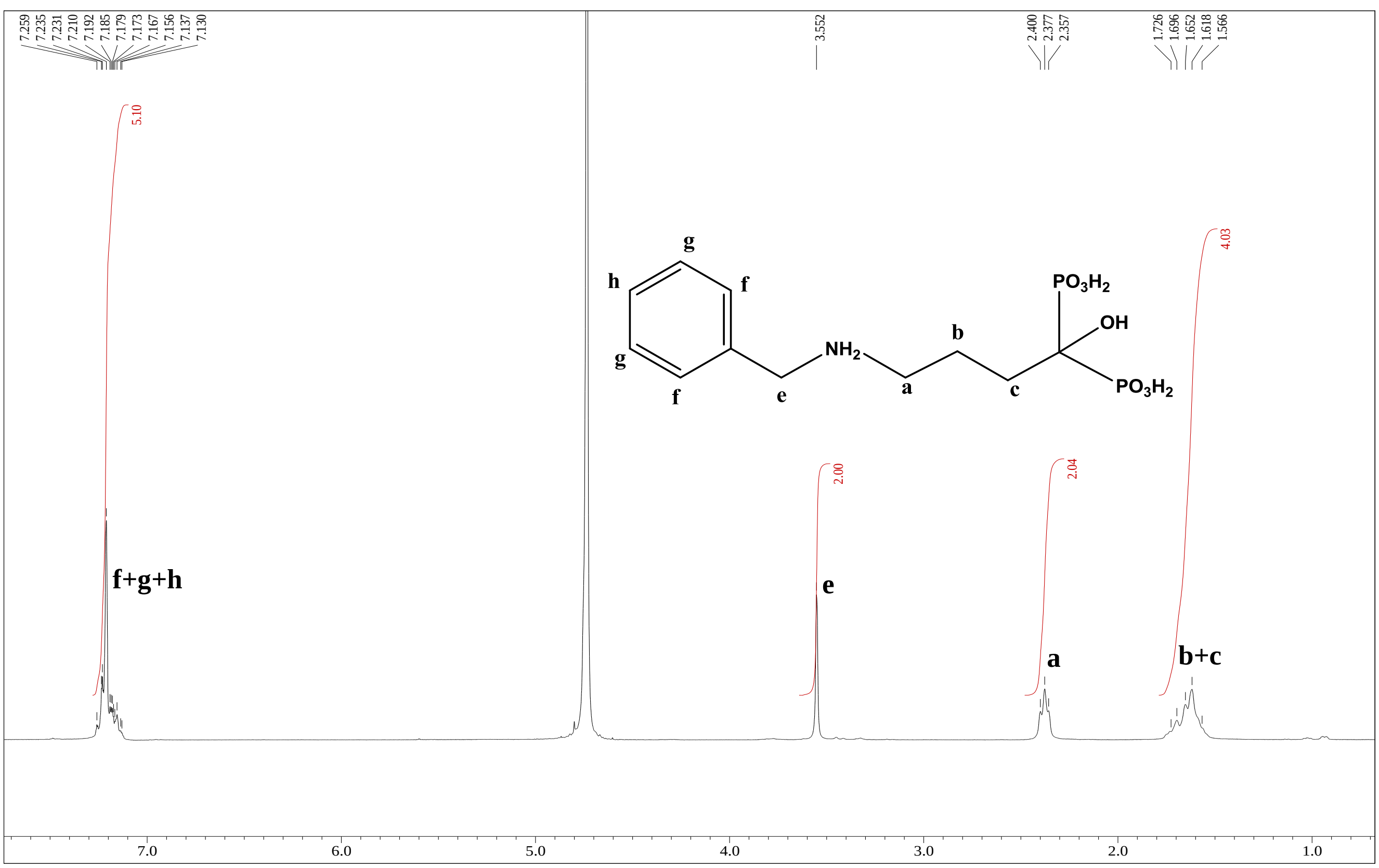

Figure S11. ^1H NMR (300 MHz) spectrum of compound **4a** in D_2O/NaOD**.**

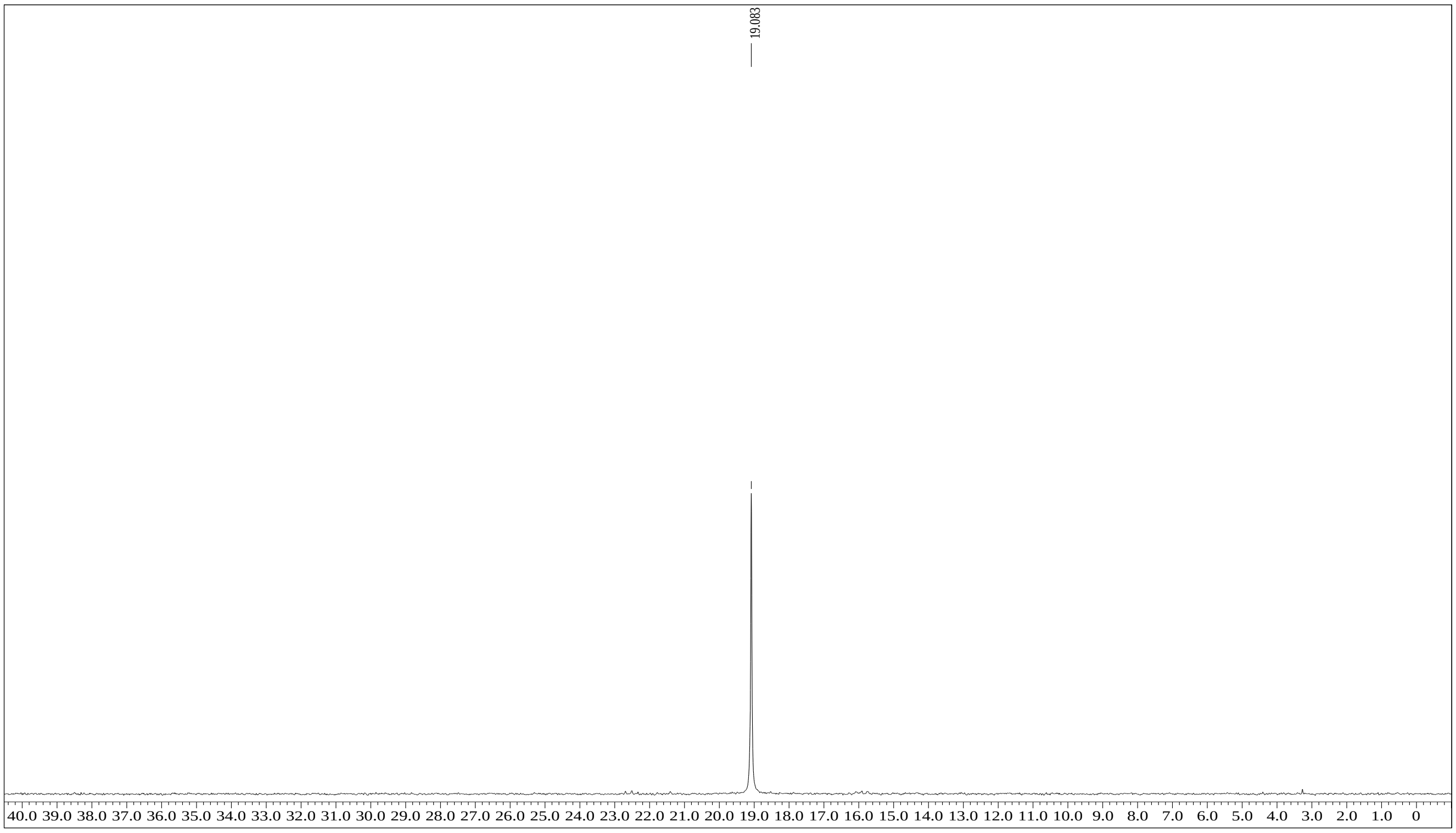

Figure S12.^{31}P{^{1}H} NMR (121 MHz) spectrum of compound **4a** in D_2O/NaOD.

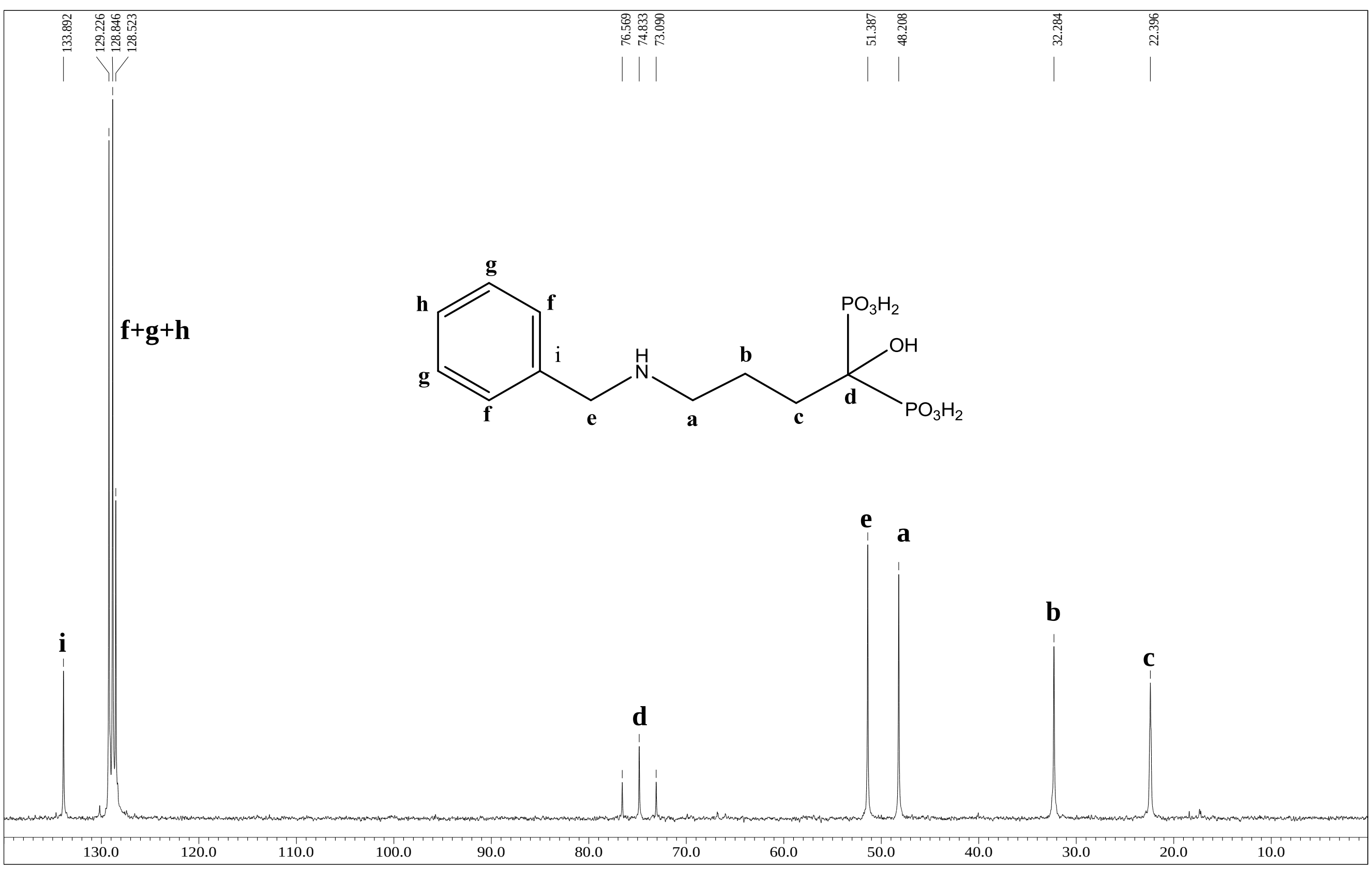

Figure S13. ^{13}C{^{1}H} NMR (75 MHz) spectrum of compound **4a** in D_2O/NaOD.

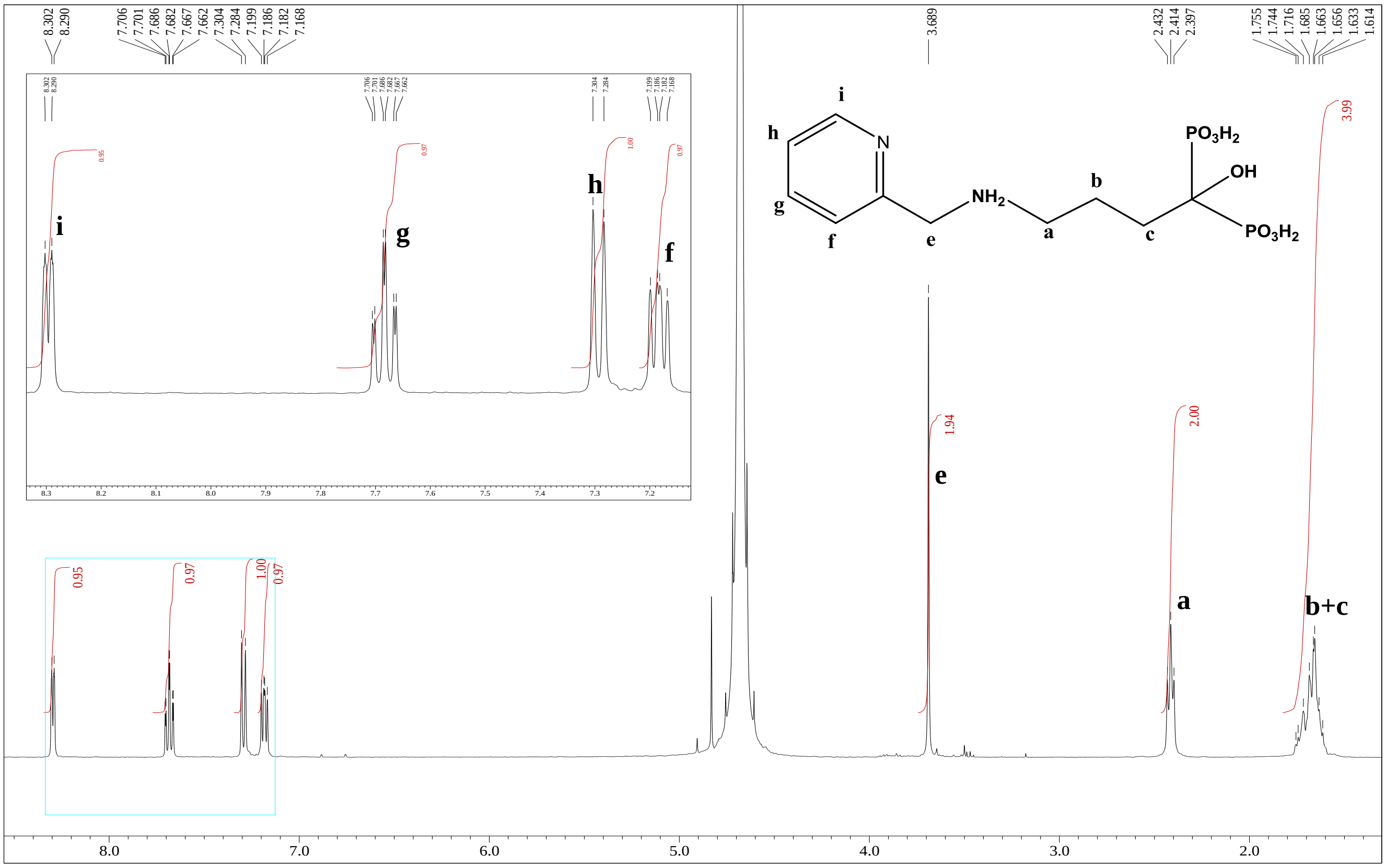

Figure S14. ^{1}H NMR (400 MHz)spectrum of compound **4b** in D_2O/NaOD.

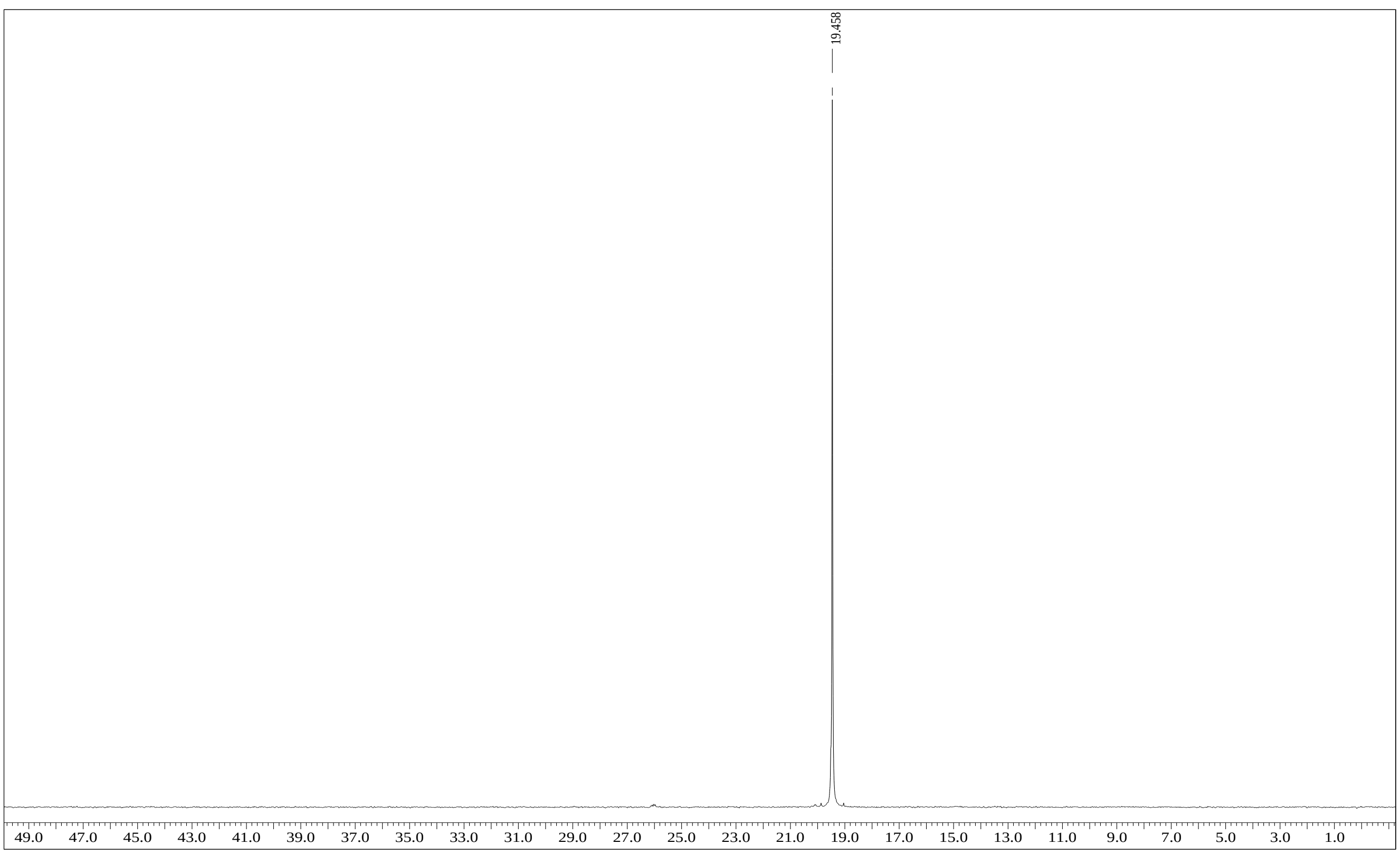

Figure S15. ^{31}P{^{1}H} NMR (162 MHz) spectrum of compound **4b** in D_2O/NaOD.

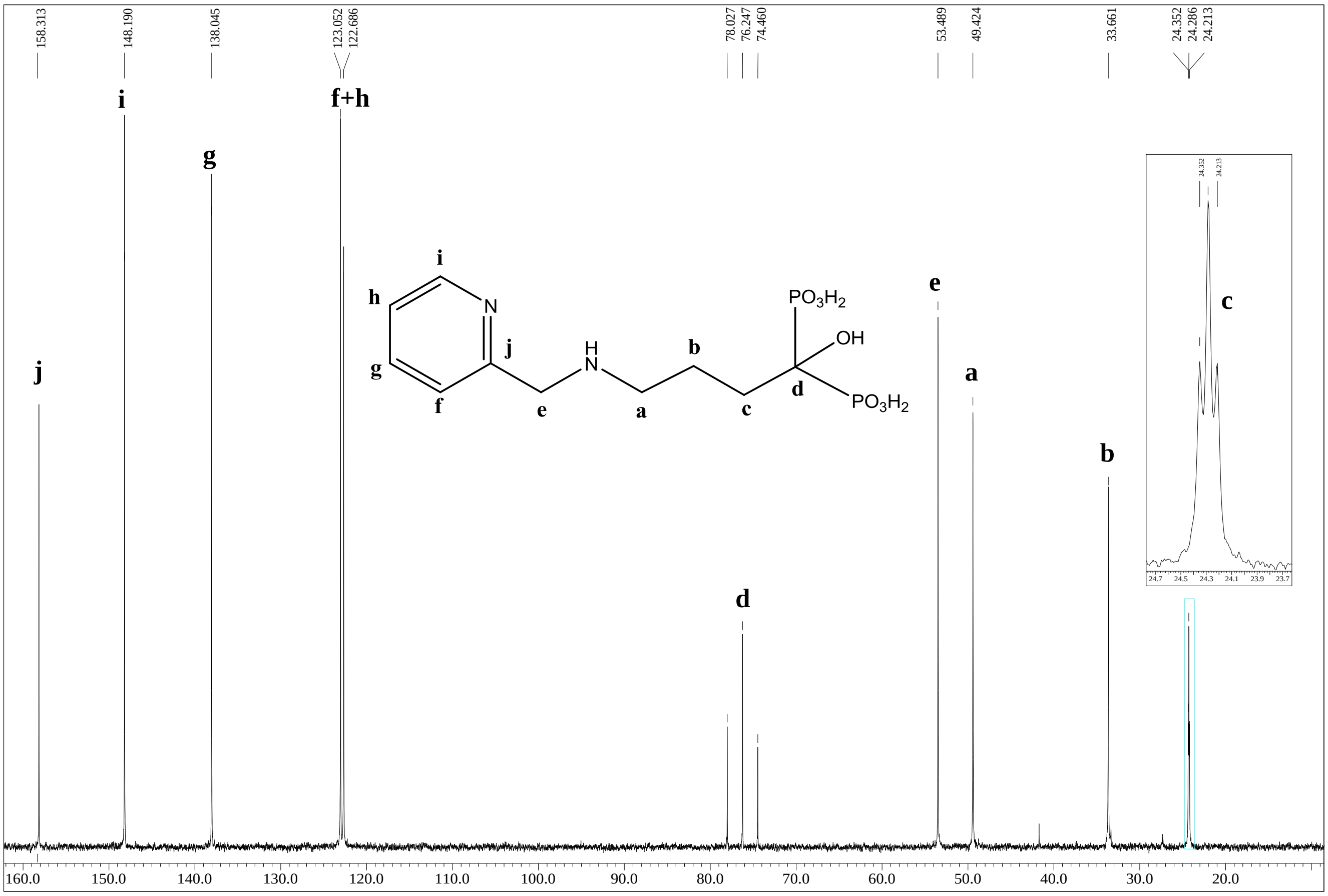

Figure S16. $^{13}C\{^1H\}$ NMR (75 MHz) spectrum of compound **4b** in D_2O/NaOD.

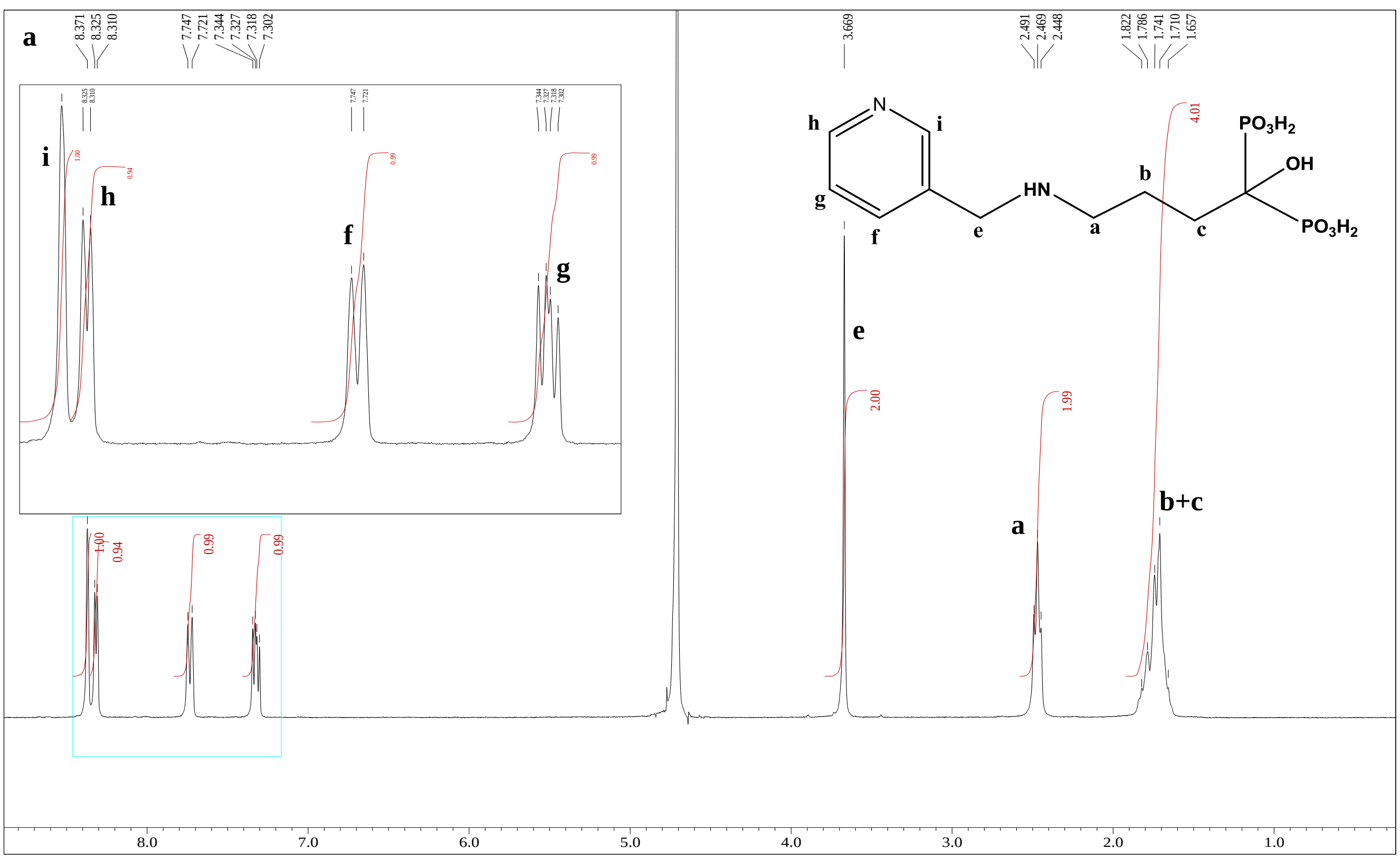

Figure S17. ^1H NMR (300 MHz) spectrum of compound **4c** in D_2O/NaOD.

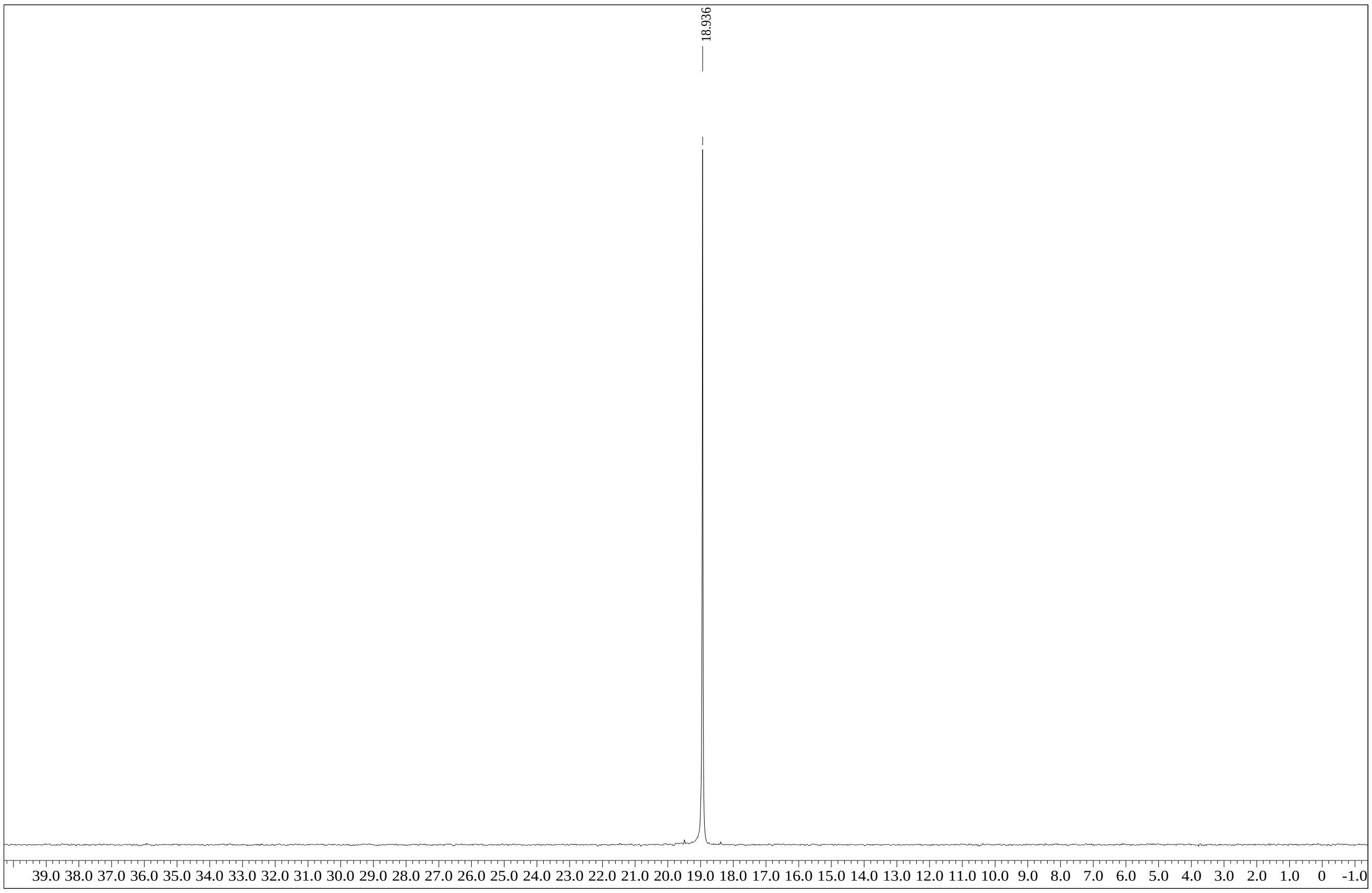

Figure S18. $^{31}P\{^1H\}$ NMR (121 MHz) spectrum of compound **4c** in D_2O/NaOD.

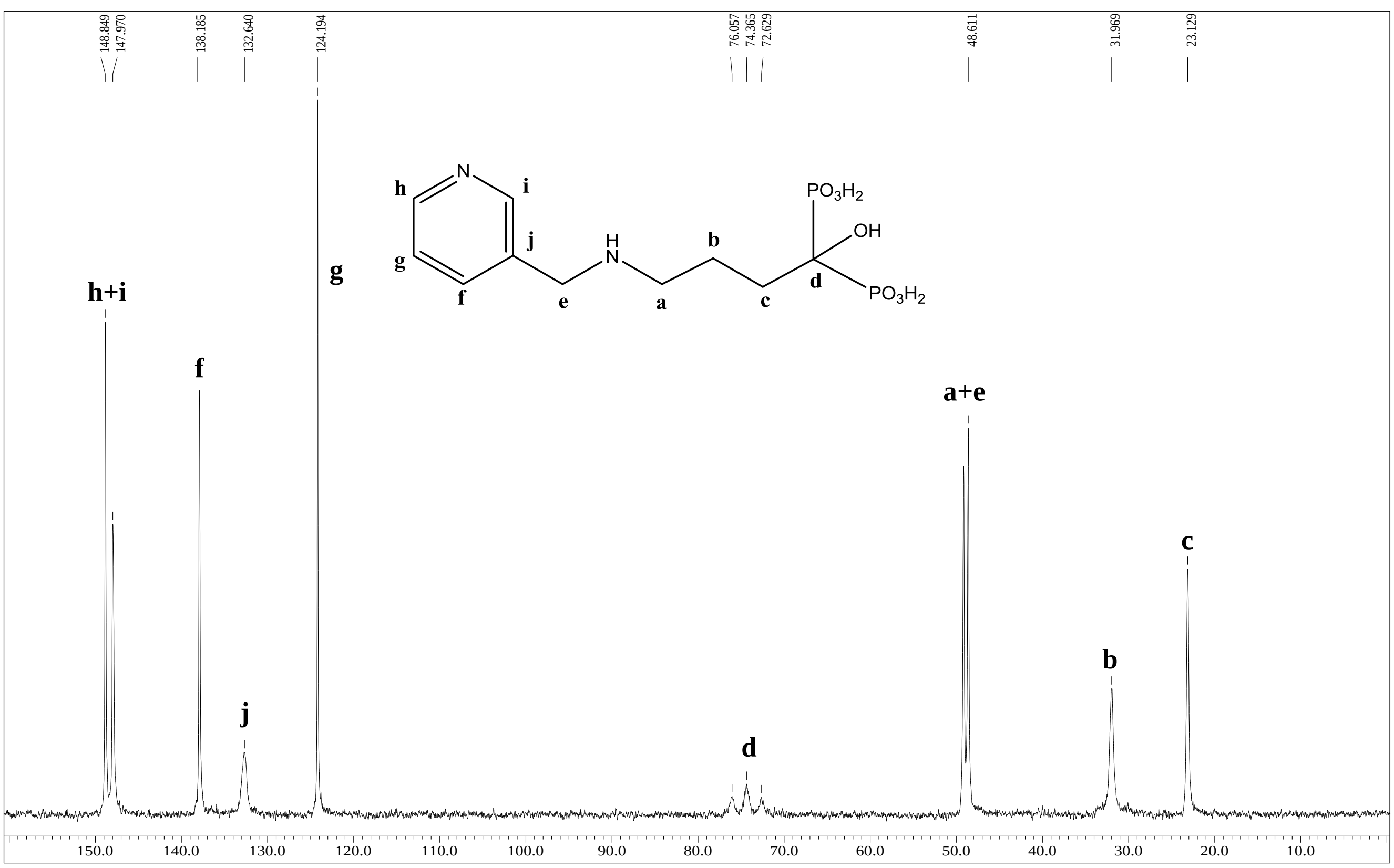

Figure S19. $^{13}C\{^1H\}$ NMR (75 MHz) spectrum of compound **4c** in D_2O/NaOD.

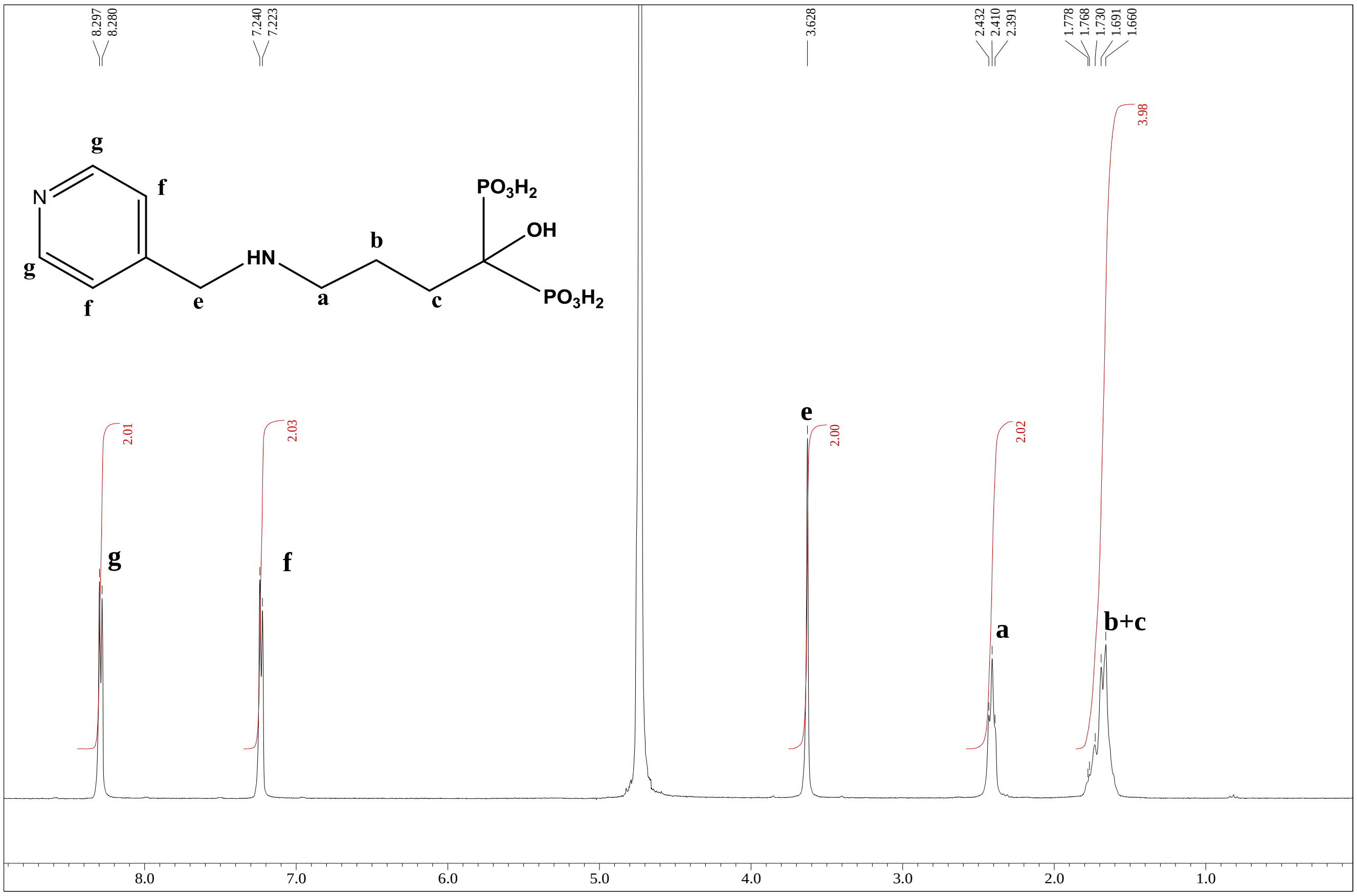

Figure S20. ^{1}H NMR (300 MHz) spectrum of compound **4d** in D_2O/NaOD.

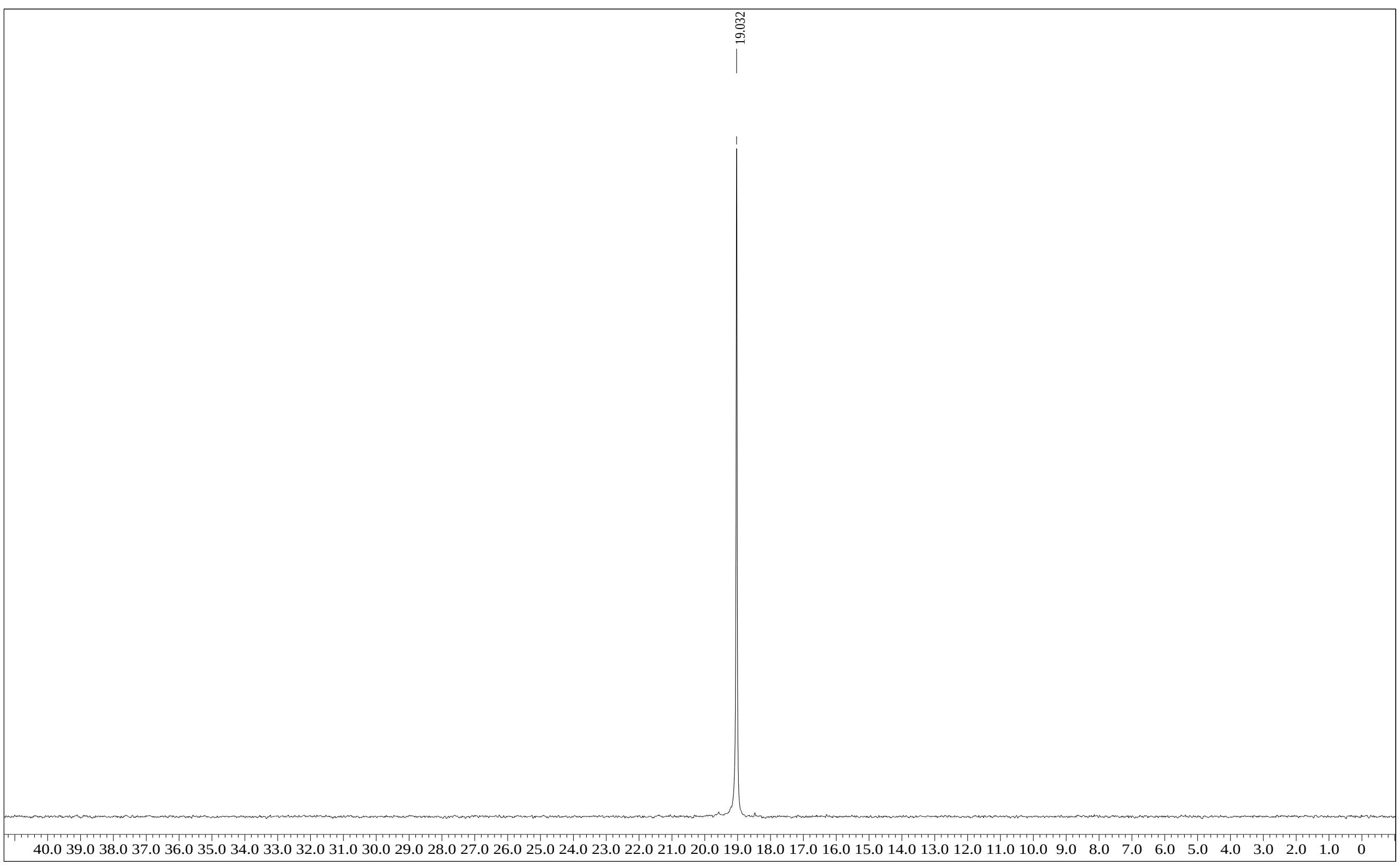

Figure S21. ^{31}P{^{1}H} NMR (121 MHz) spectrum of compound **4d** in D_2O/NaOD.

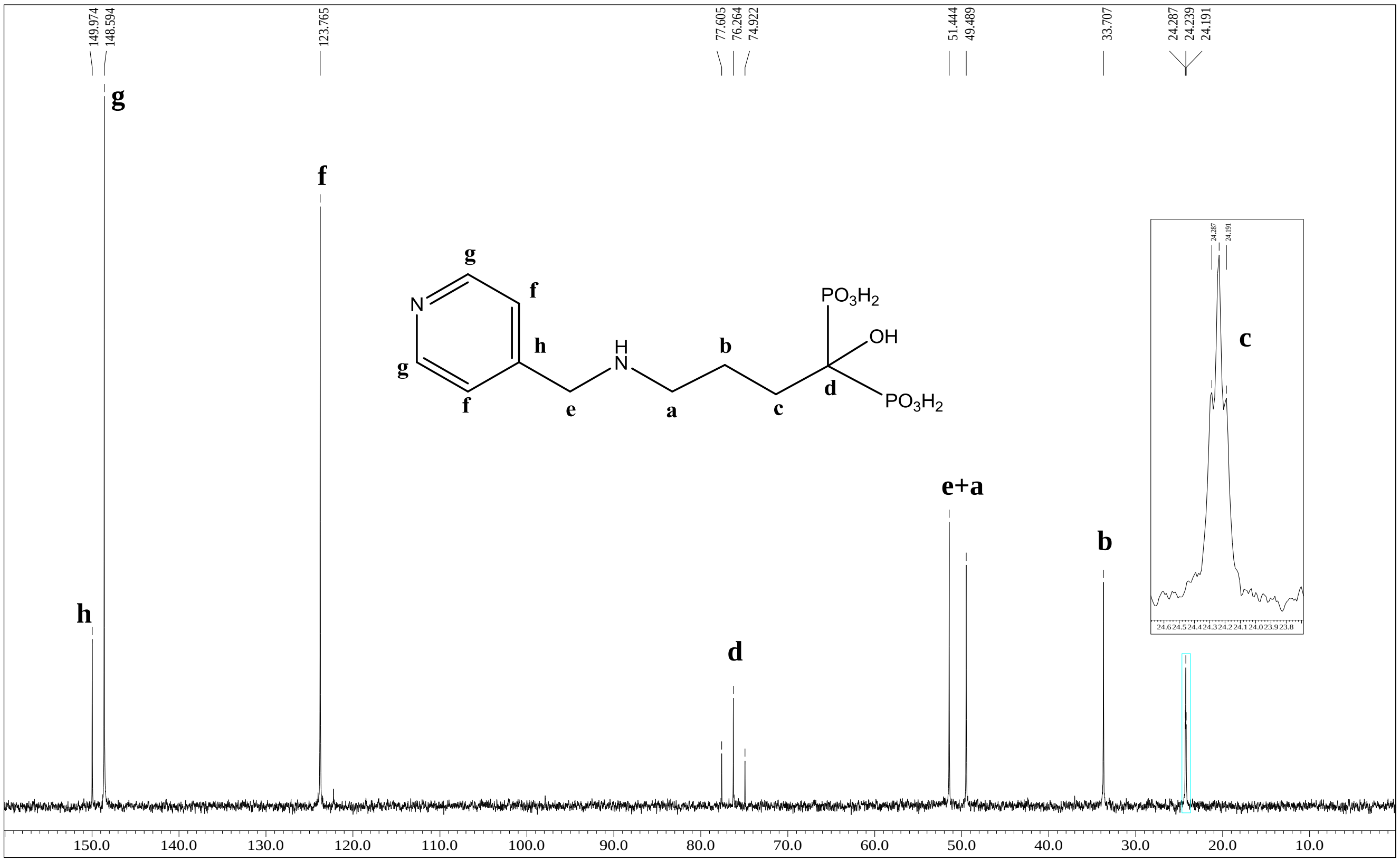

Figure S22. ^{13}C{^{1}H} NMR (100 MHz) spectrum of compound **4d** in D_2O/NaOD.

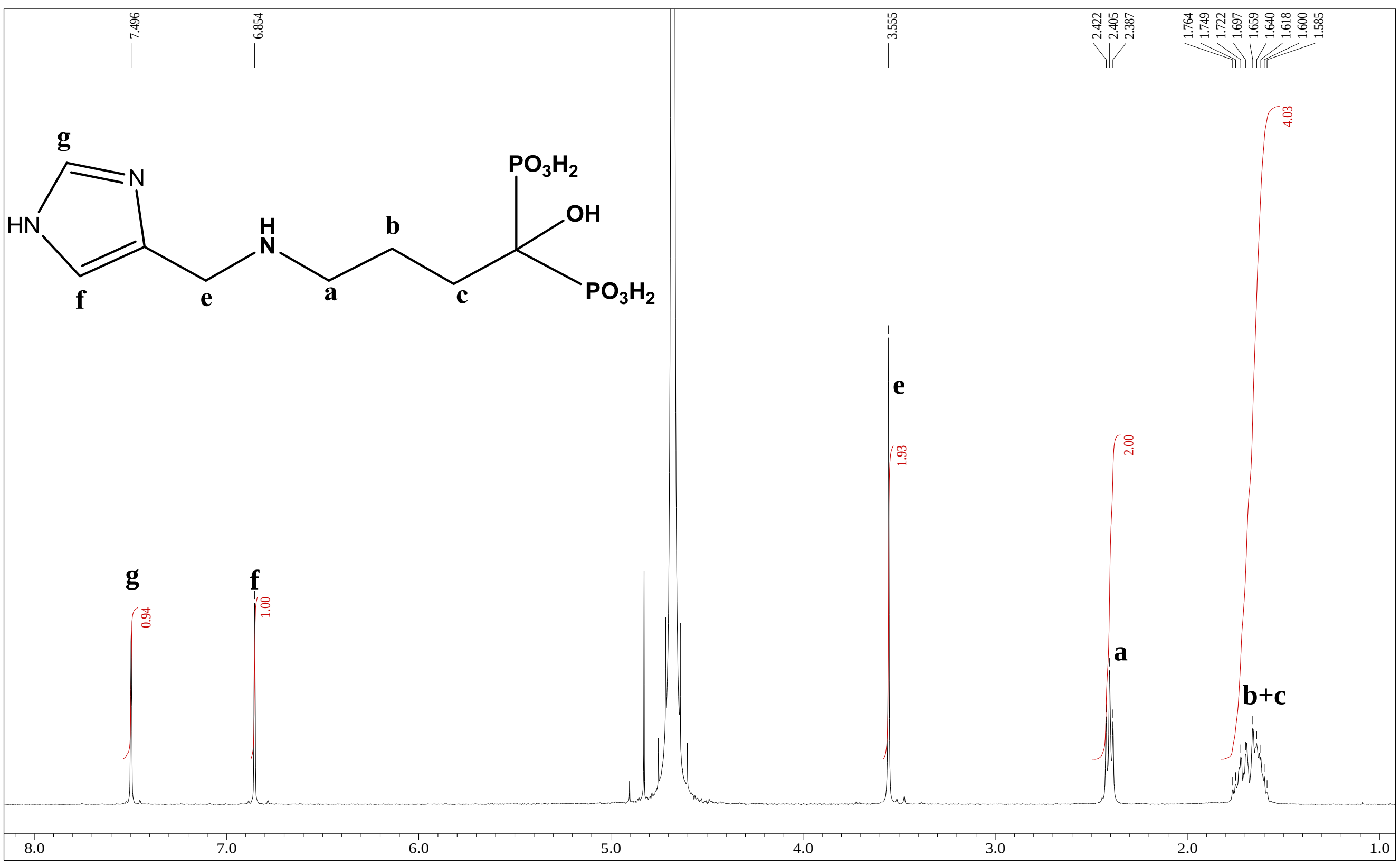

Figure S23. ^{1}H NMR (400 MHz)spectrum of compound **4e** in D_2O/NaOD.

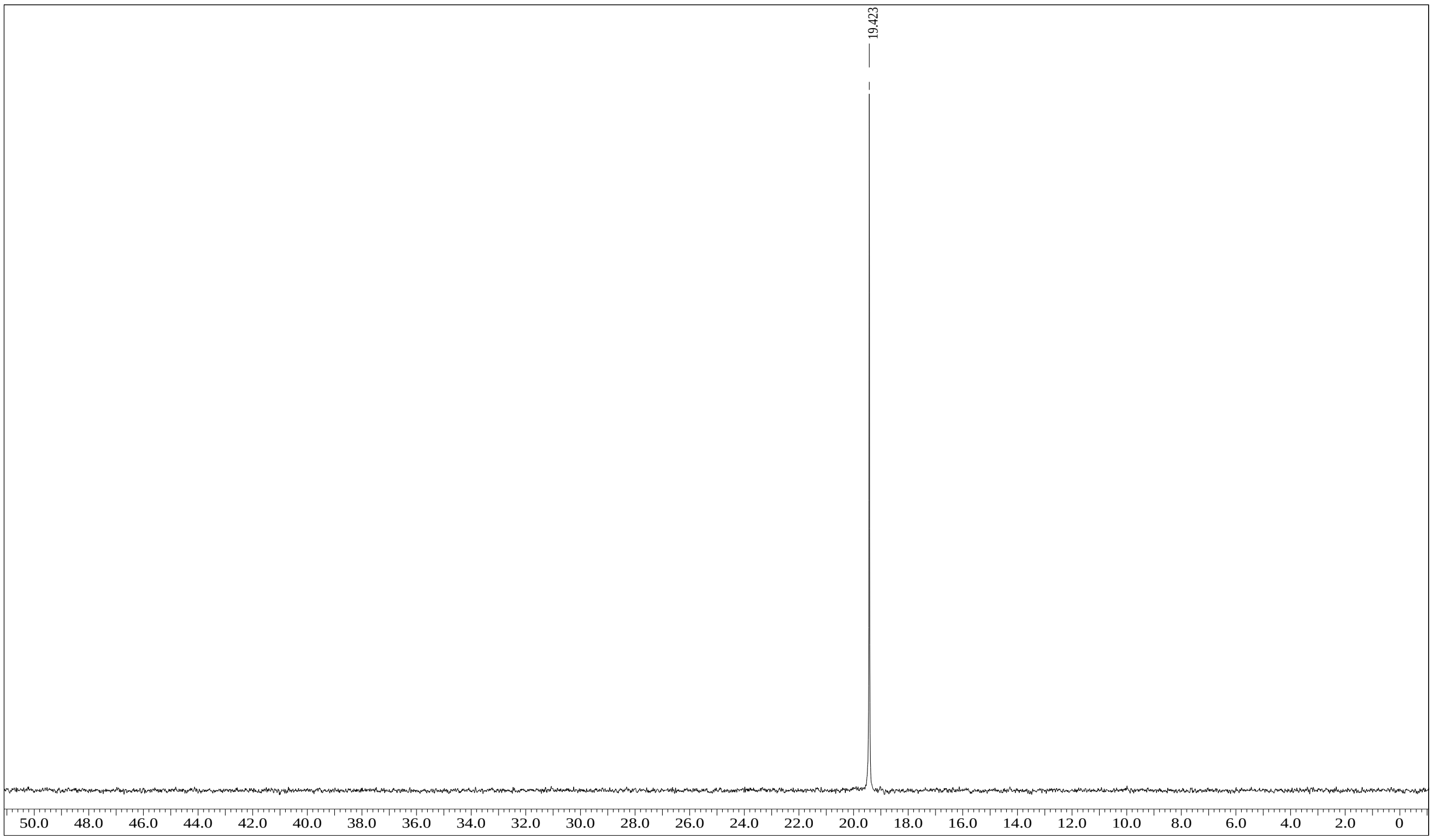

Figure S24. $^{31}P\{^1H\}$ NMR (162 MHz) spectrum of compound **4e** in D_2O/NaOD.

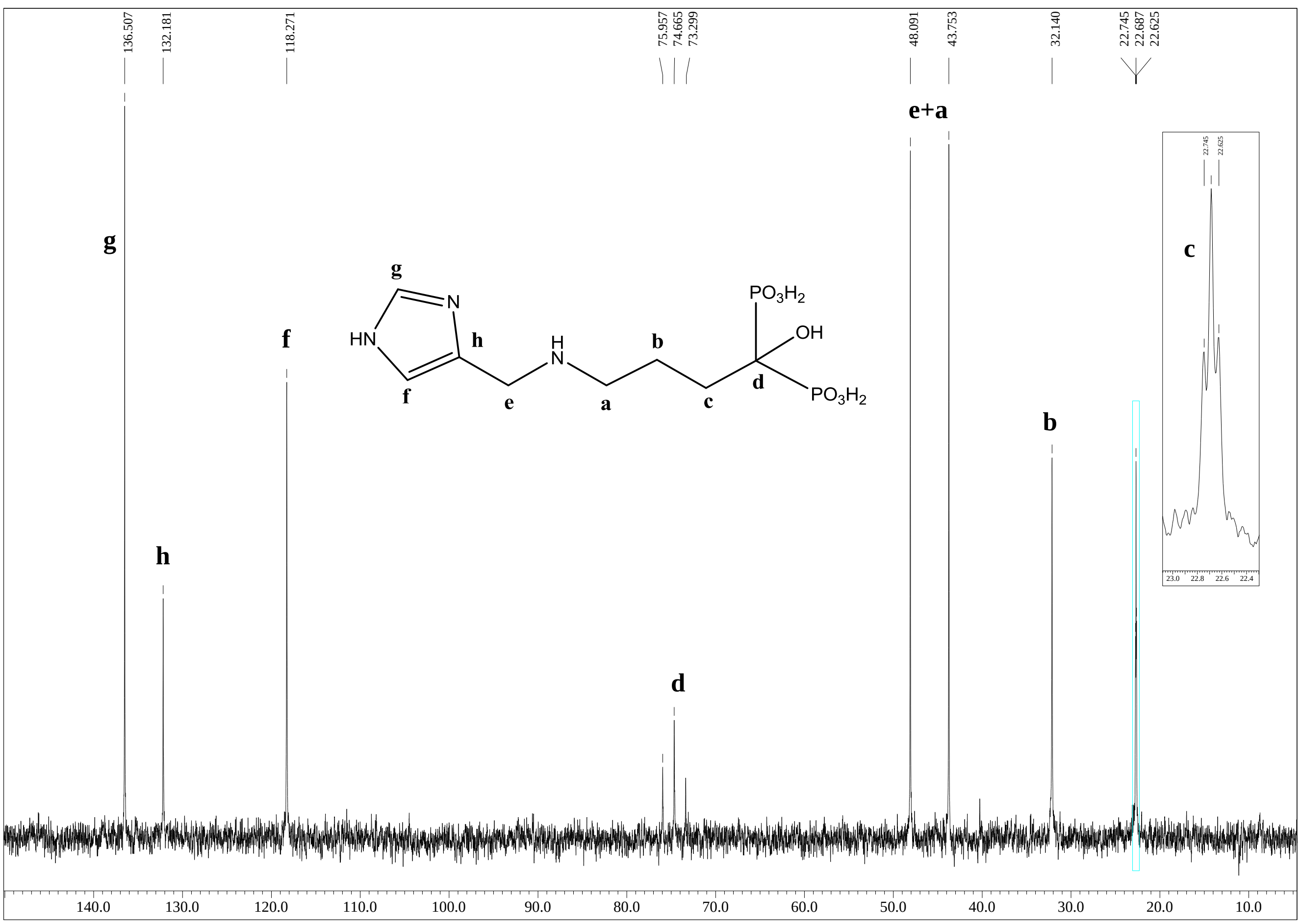

Figure S25. $^{13}C\{^1H\}$ NMR (100 MHz) spectrum of compound **4e** in D_2O/NaOD.

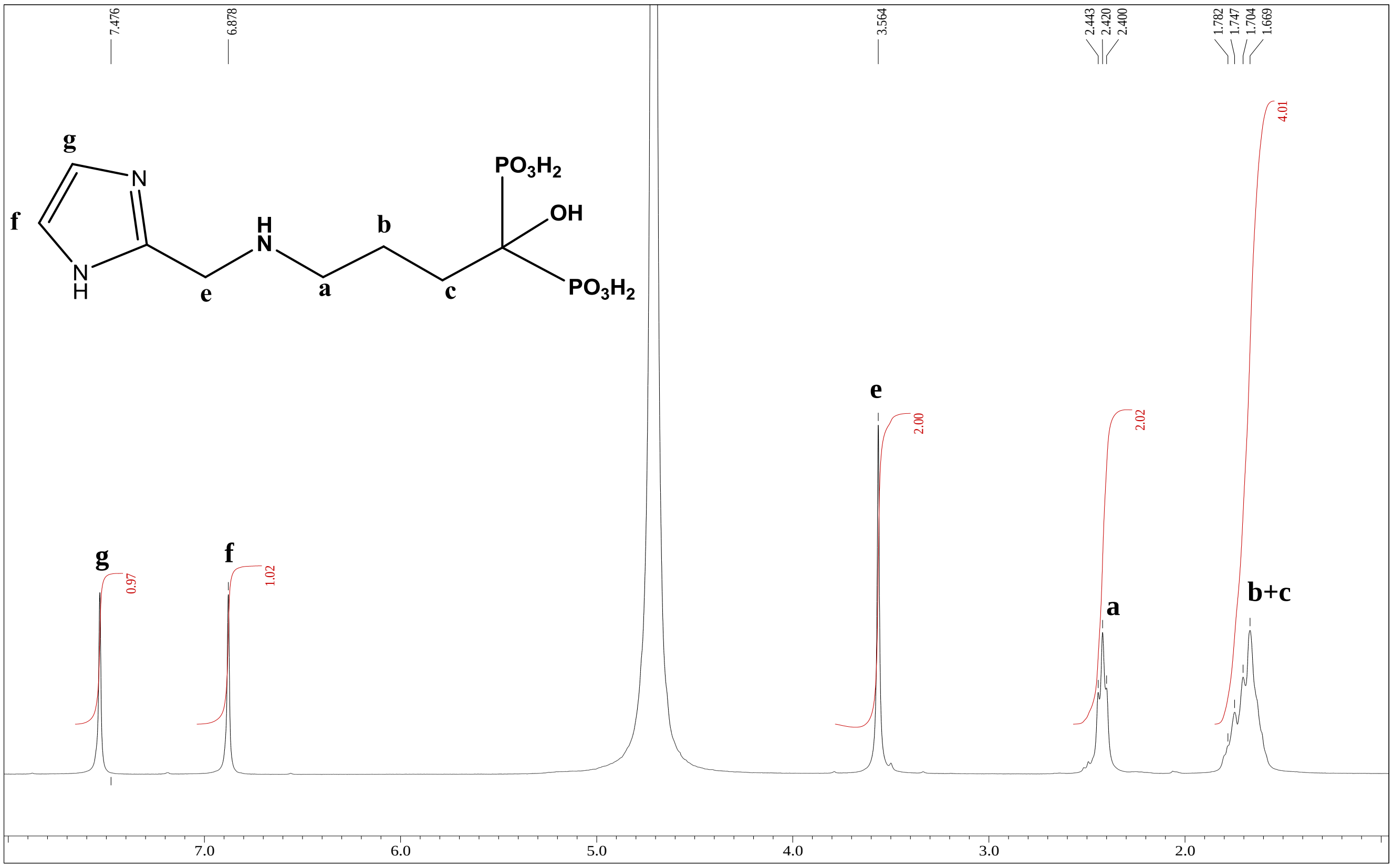

Figure S26. ^{1}H NMR (300 MHz) spectrum of compound **4f** in D_2O/NaOD.

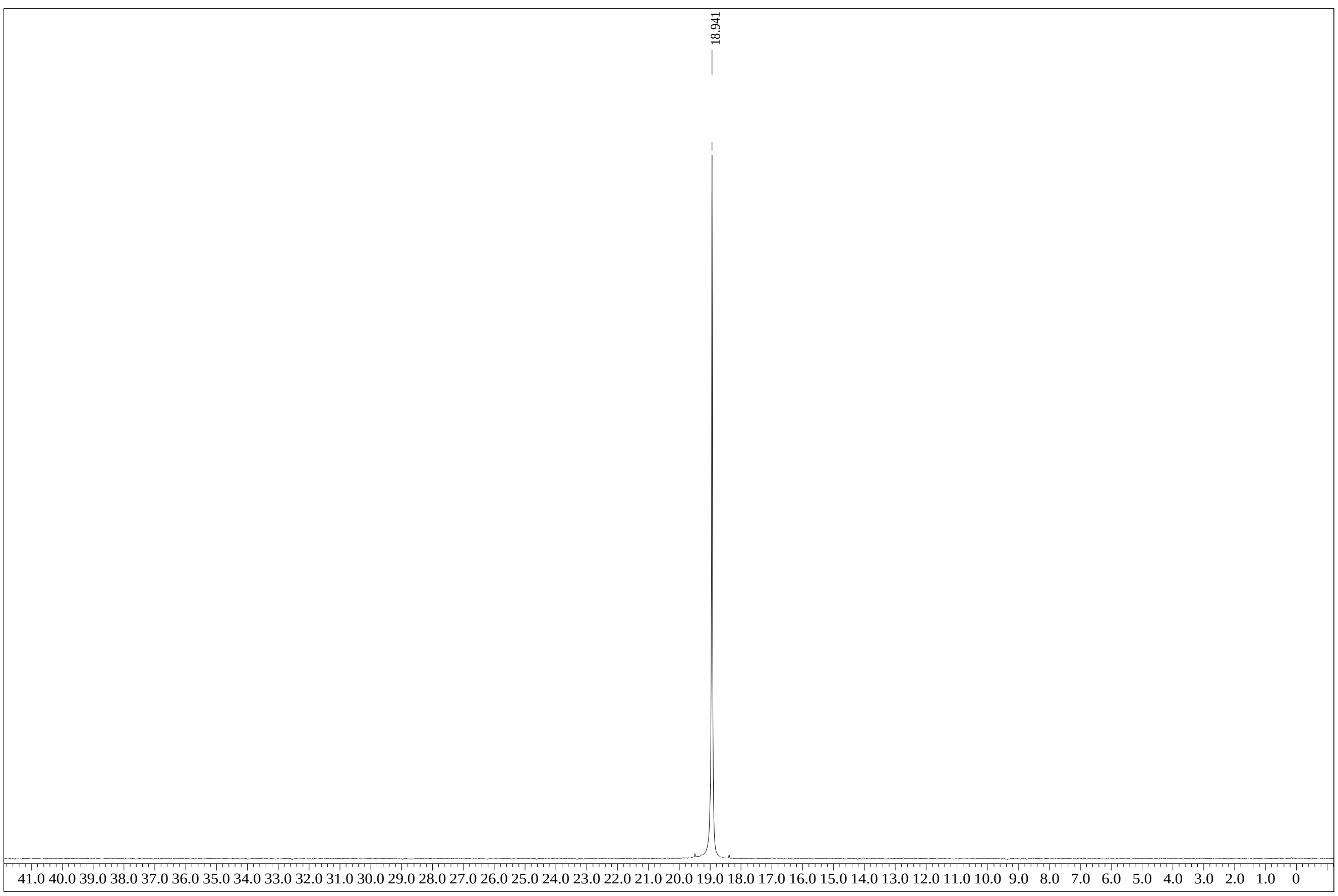

Figure S27. ^{31}P{^{1}H} NMR (121 MHz) spectrum of compound **4f** in D_2O/NaOD.

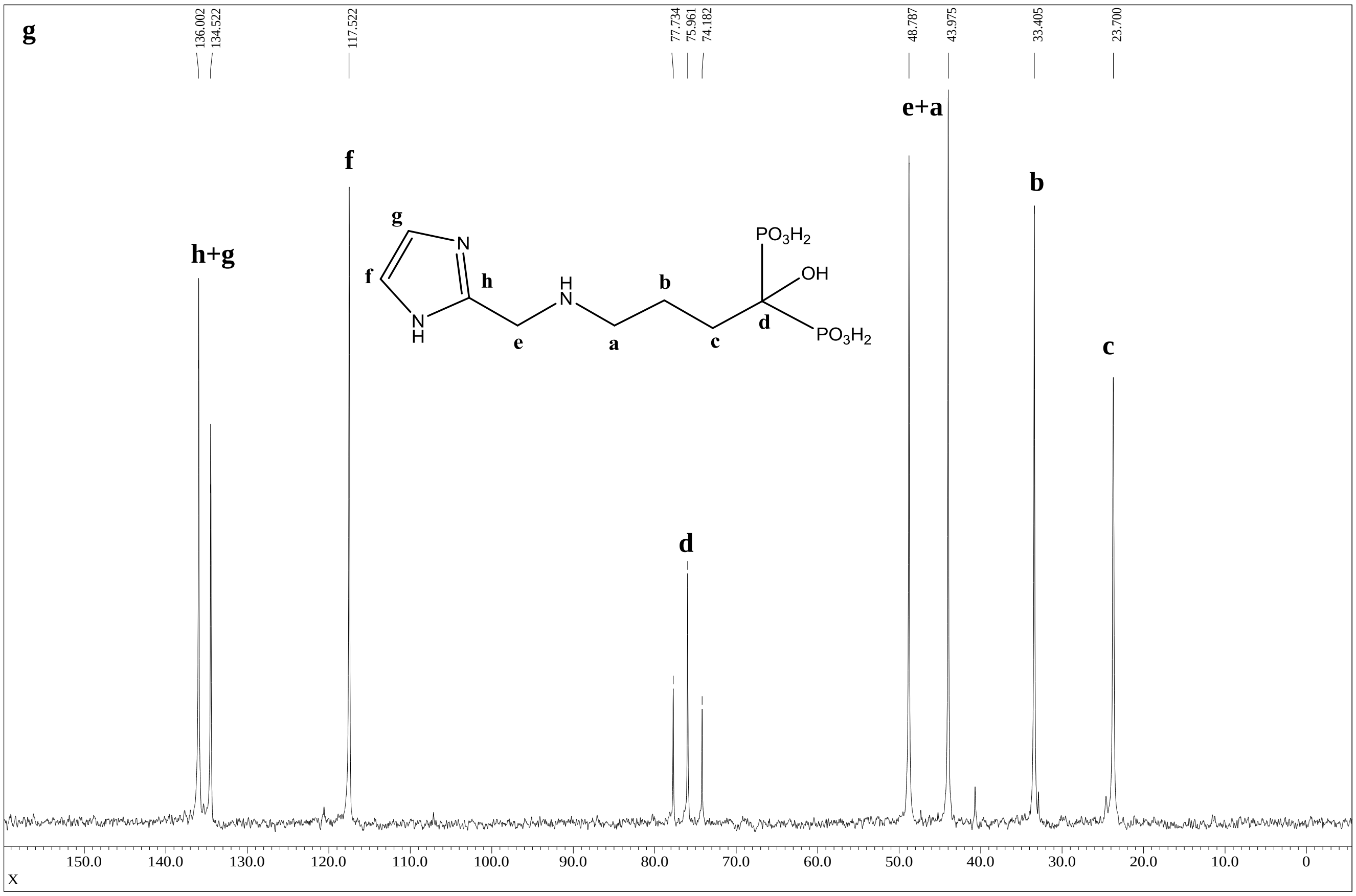

Figure S28. $^{13}C\{^{1}H\}$ NMR (75 MHz) spectrum of compound **4f** in D_2O/NaOD.

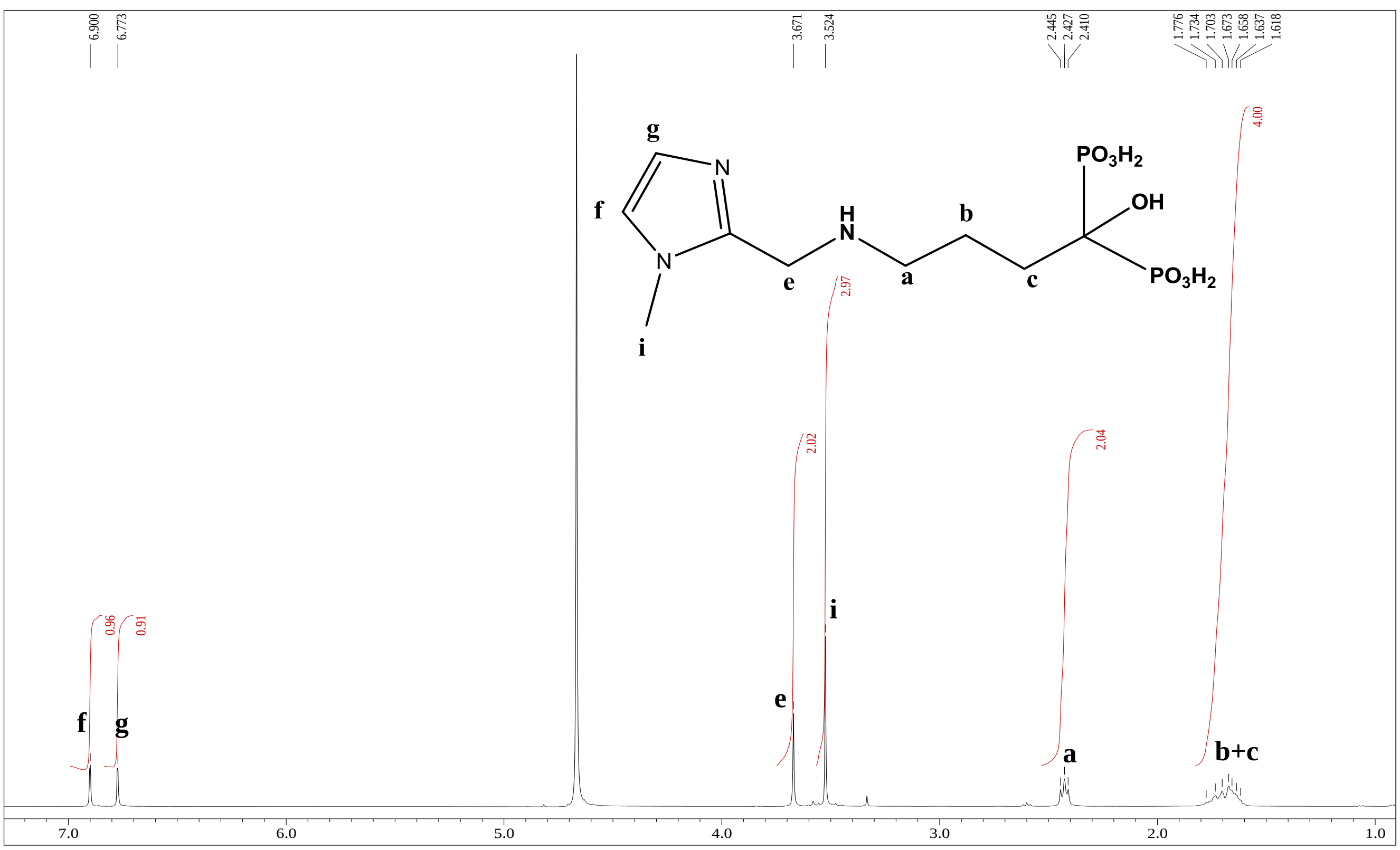

Figure S29. ^{1}H NMR (400 MHz) spectrum of compound **4g** in D_2O/NaOD.

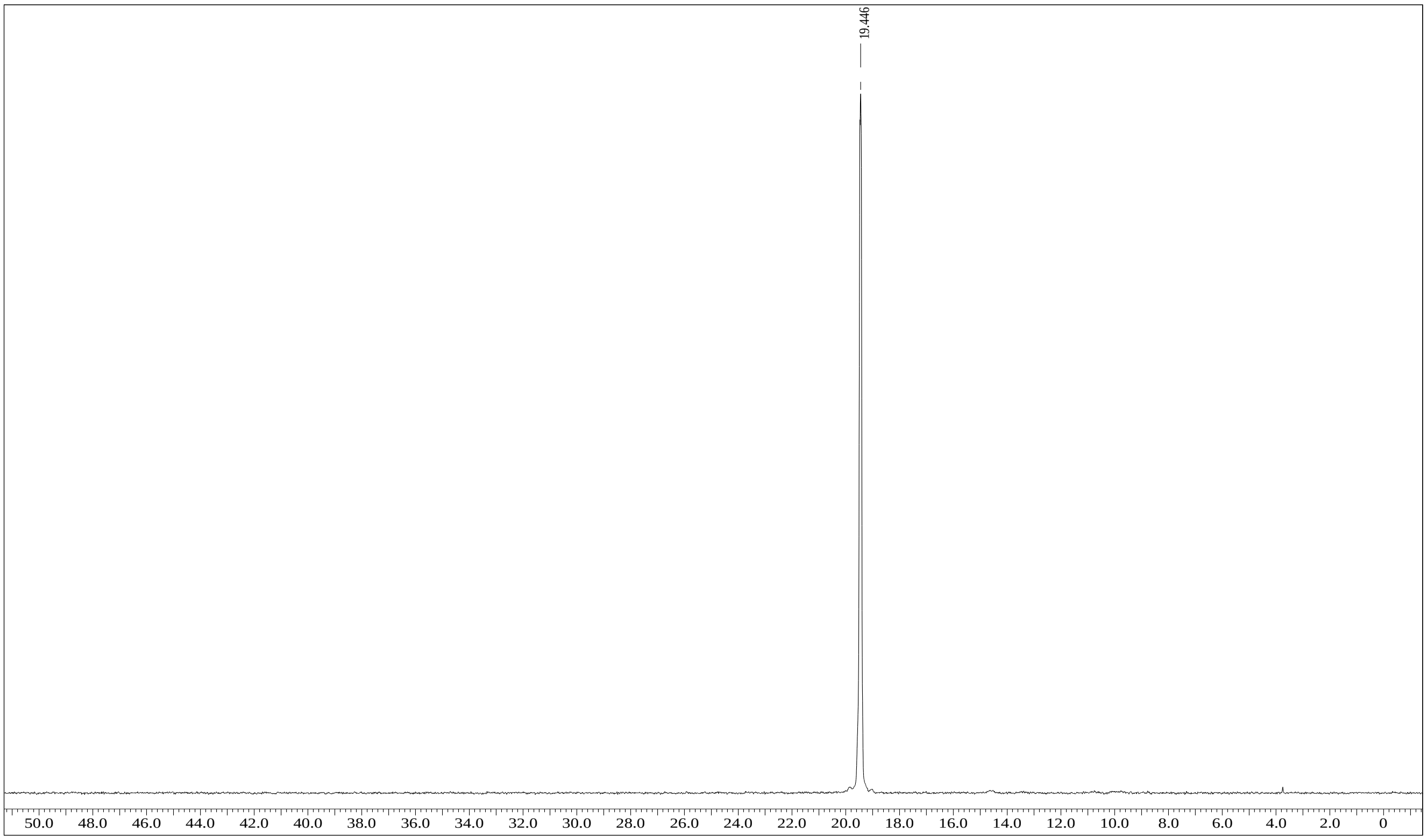

Figure S30. $^{31}P\{^{1}H\}$ NMR (162 MHz) spectrum of compound **4g** in D_2O/NaOD.

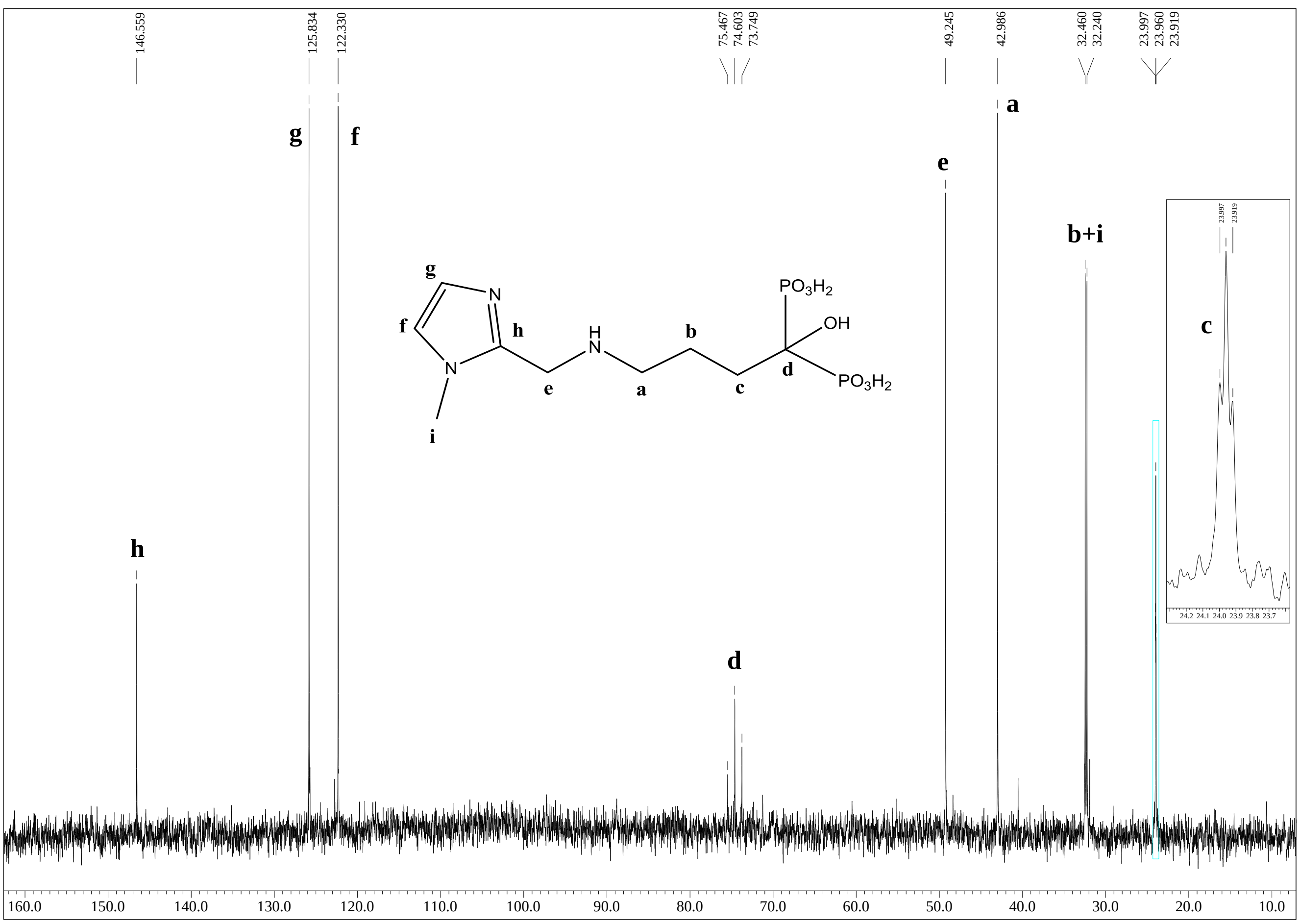

Figure S31. $^{13}C\{^{1}H\}$ NMR (150 MHz) spectrum of compound **4g** in D_2O/NaOD.

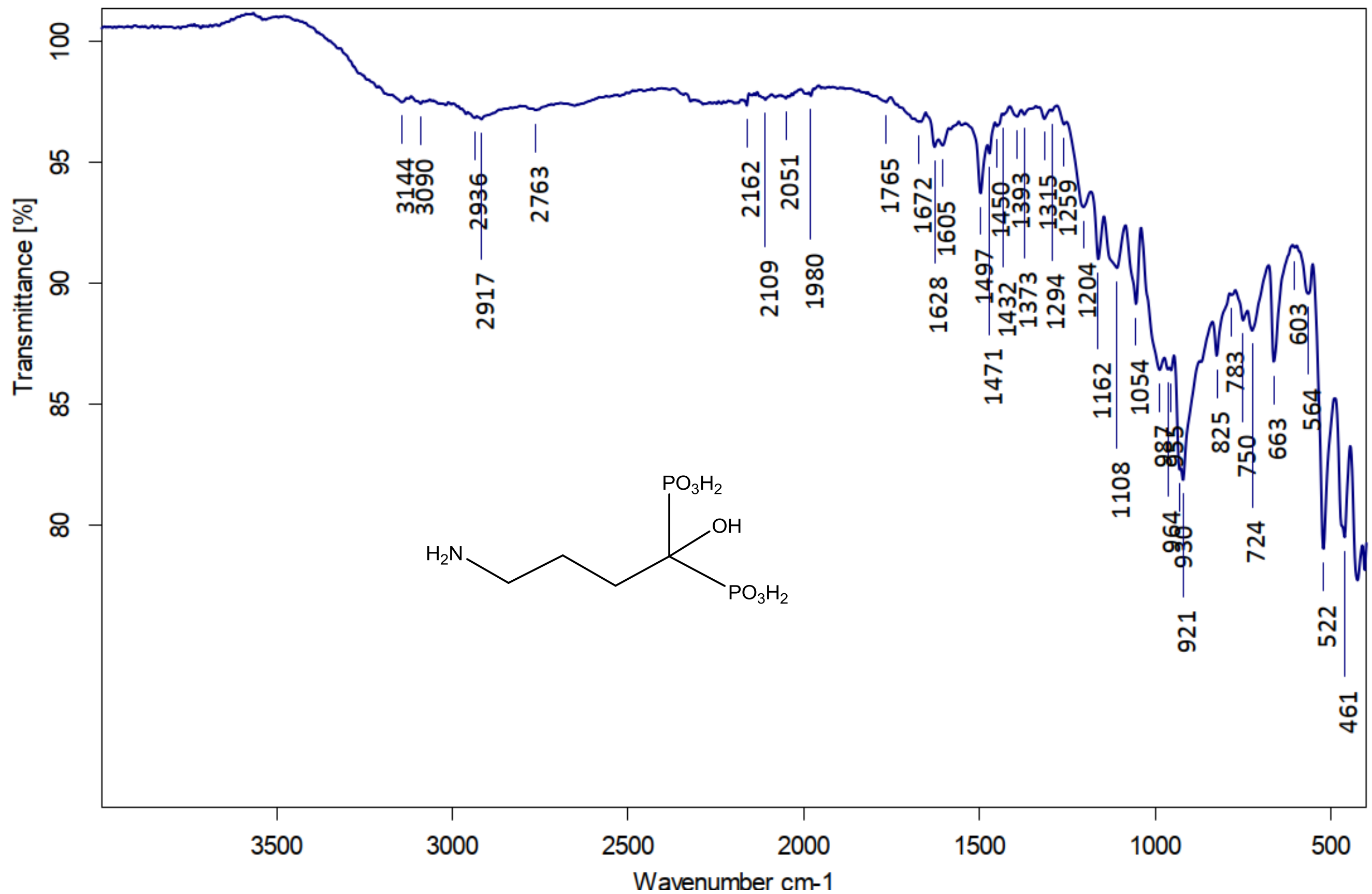

Figure S32. ATR-FTIR spectrum of Alendronic acid **2**.

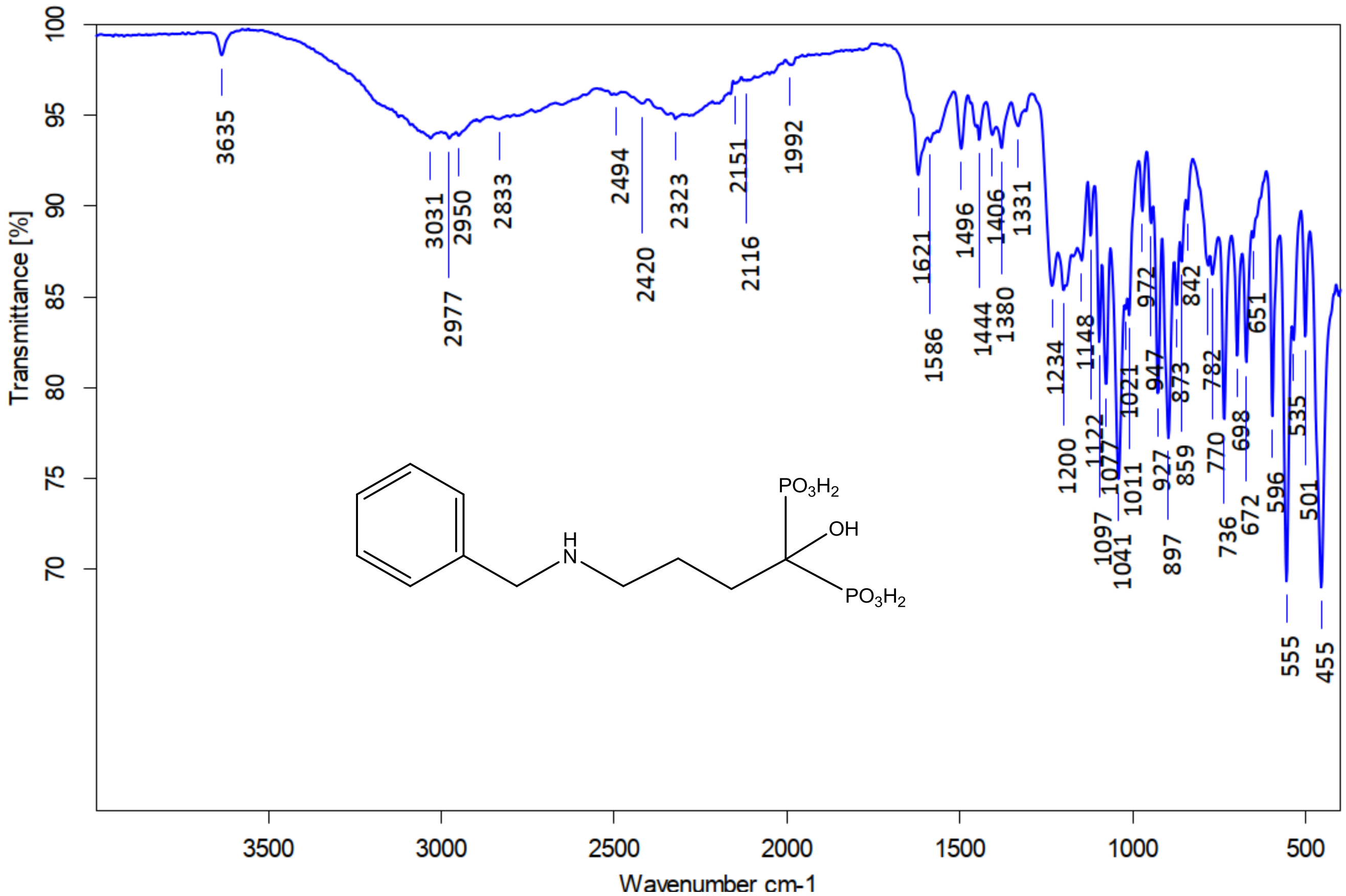

Figure S33. ATR-FTIR spectrum of compound **4a**.

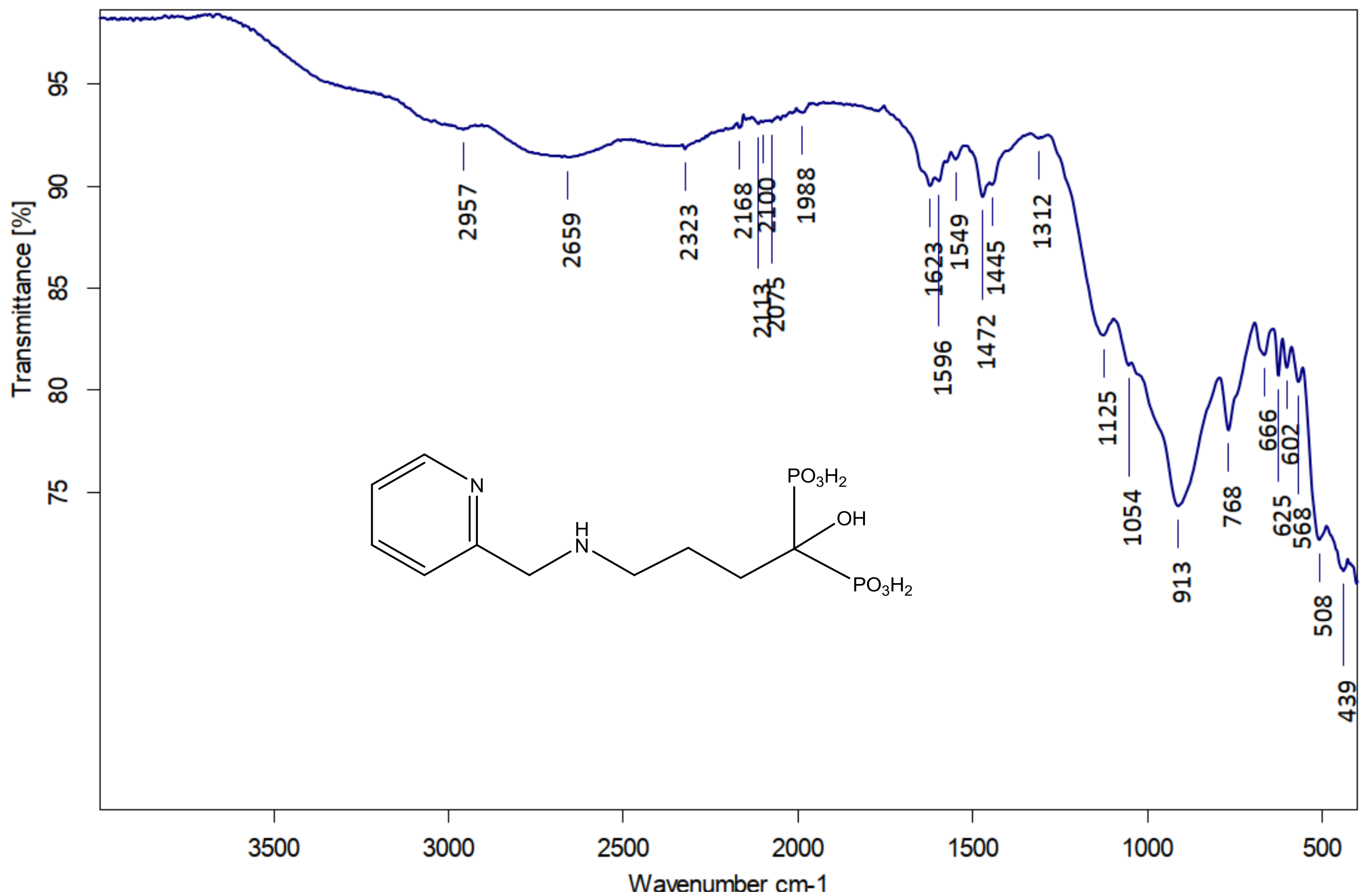

Figure S34. ATR-FTIR spectrum of compound **4b**.

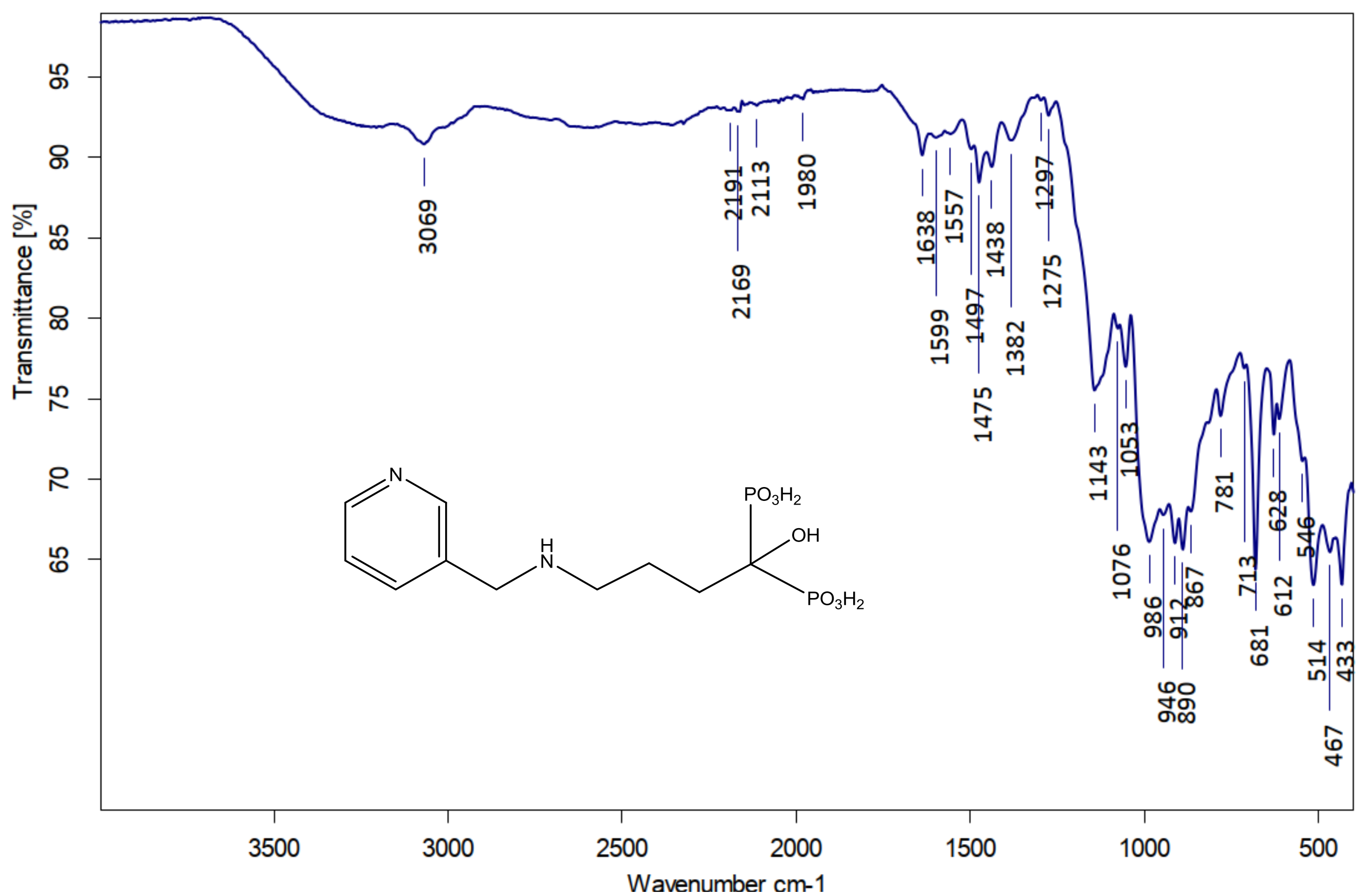

Figure S35. ATR-FTIR spectrum of compound **4c**.

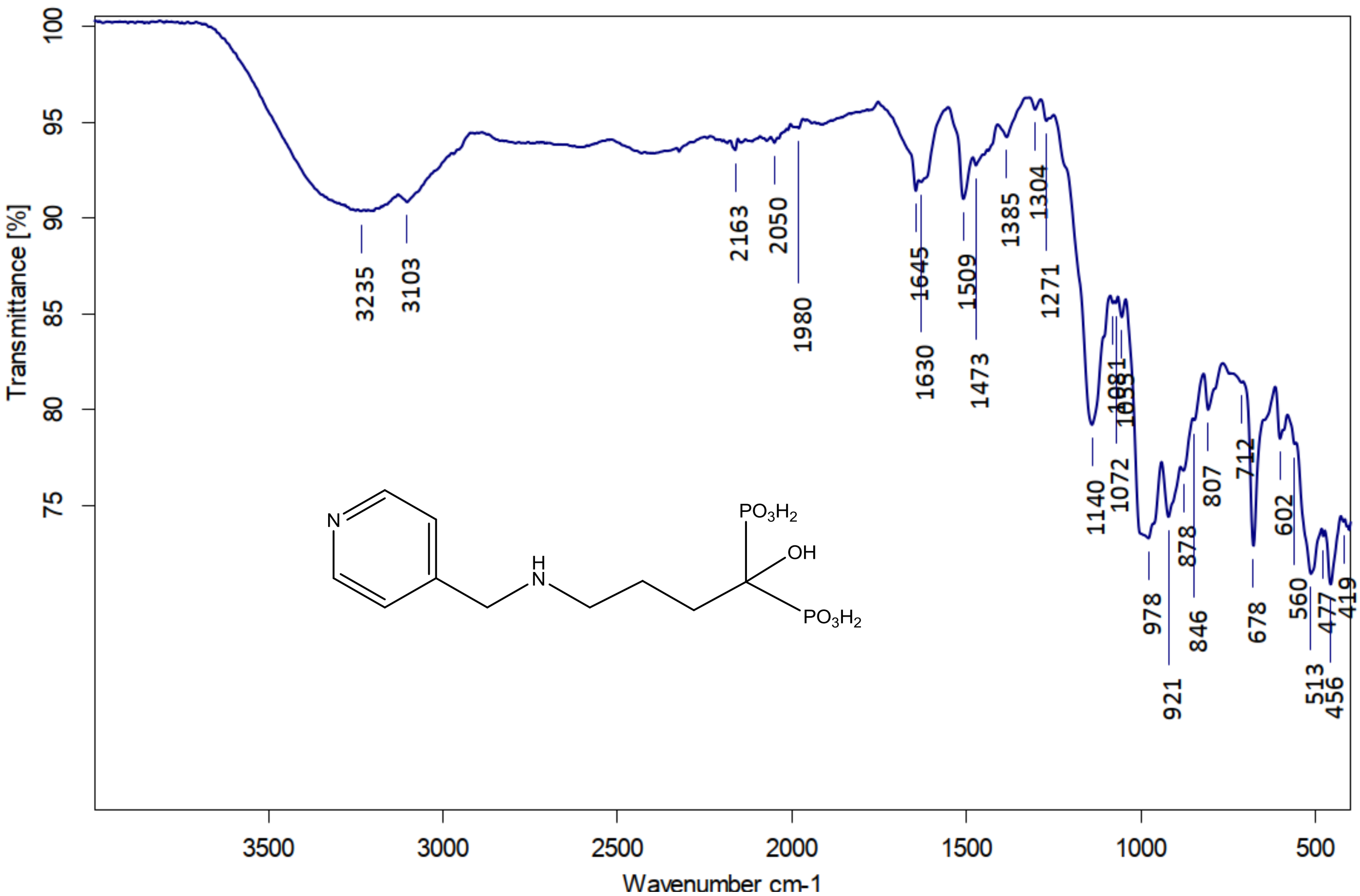

Figure S36. ATR-FTIR spectrum of compound **4d**.

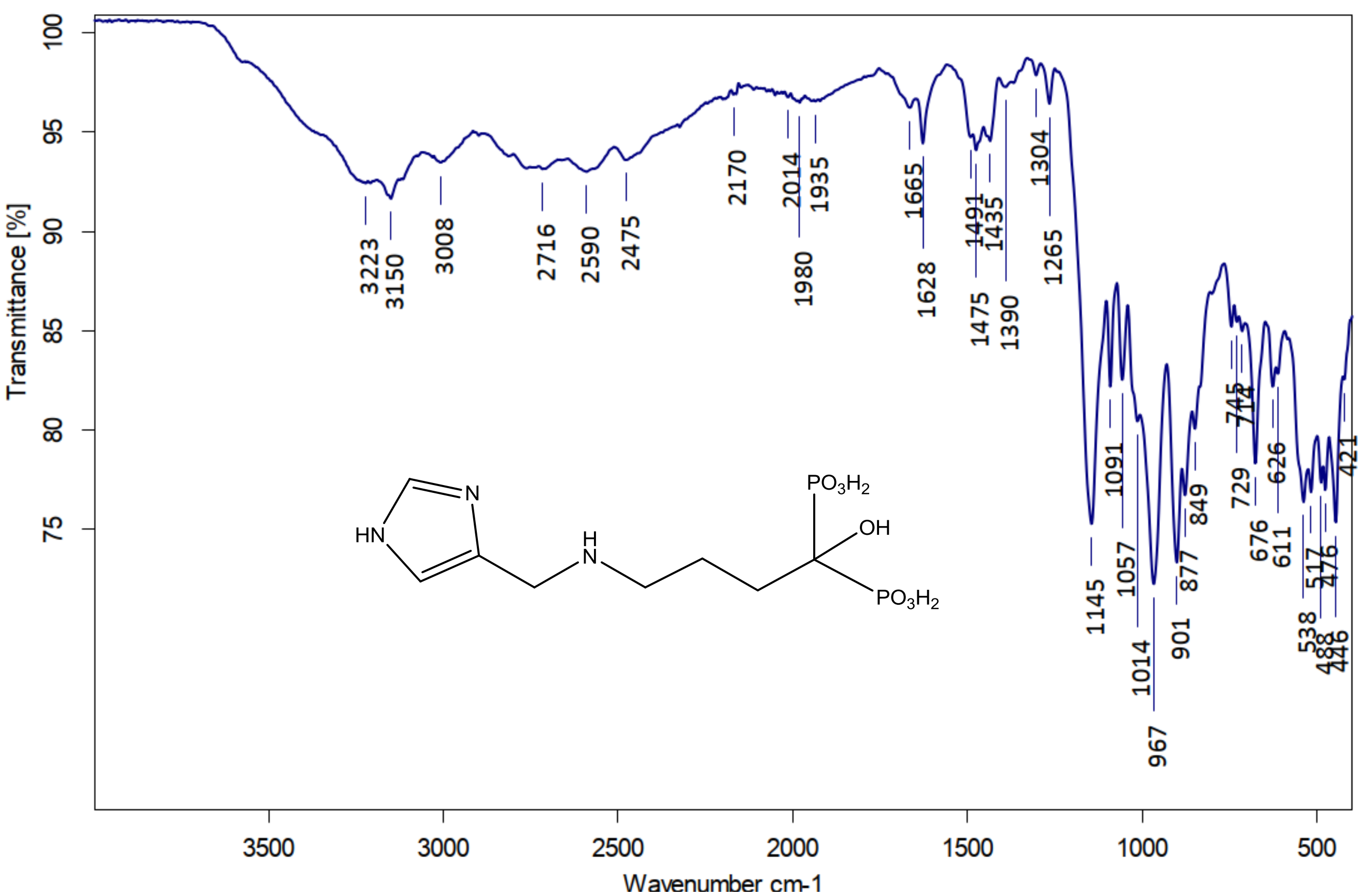

Figure S37. ATR-FTIR spectrum of compound **4e**.

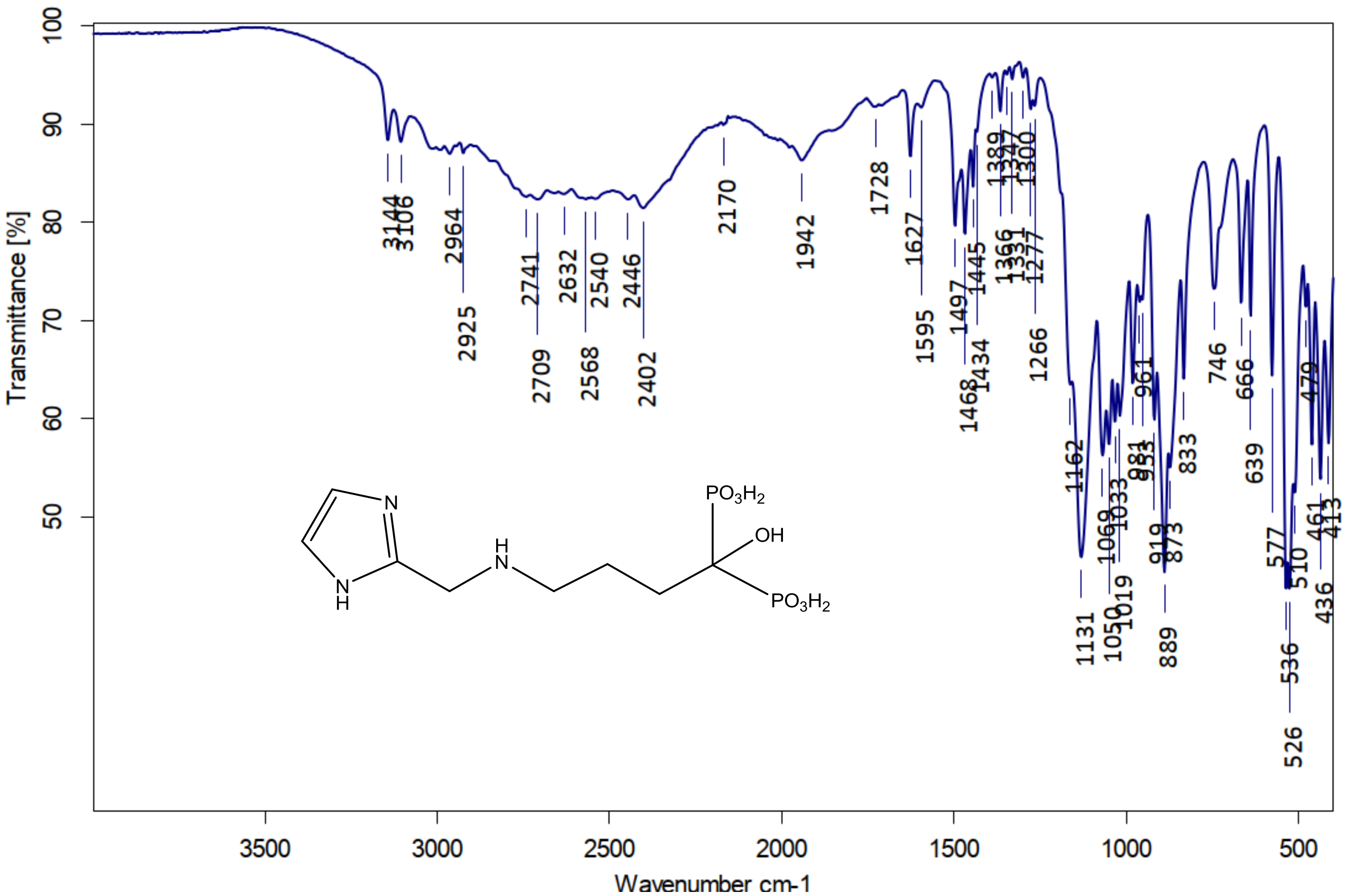

Figure S38. ATR-FTIR spectrum of compound **4f**.

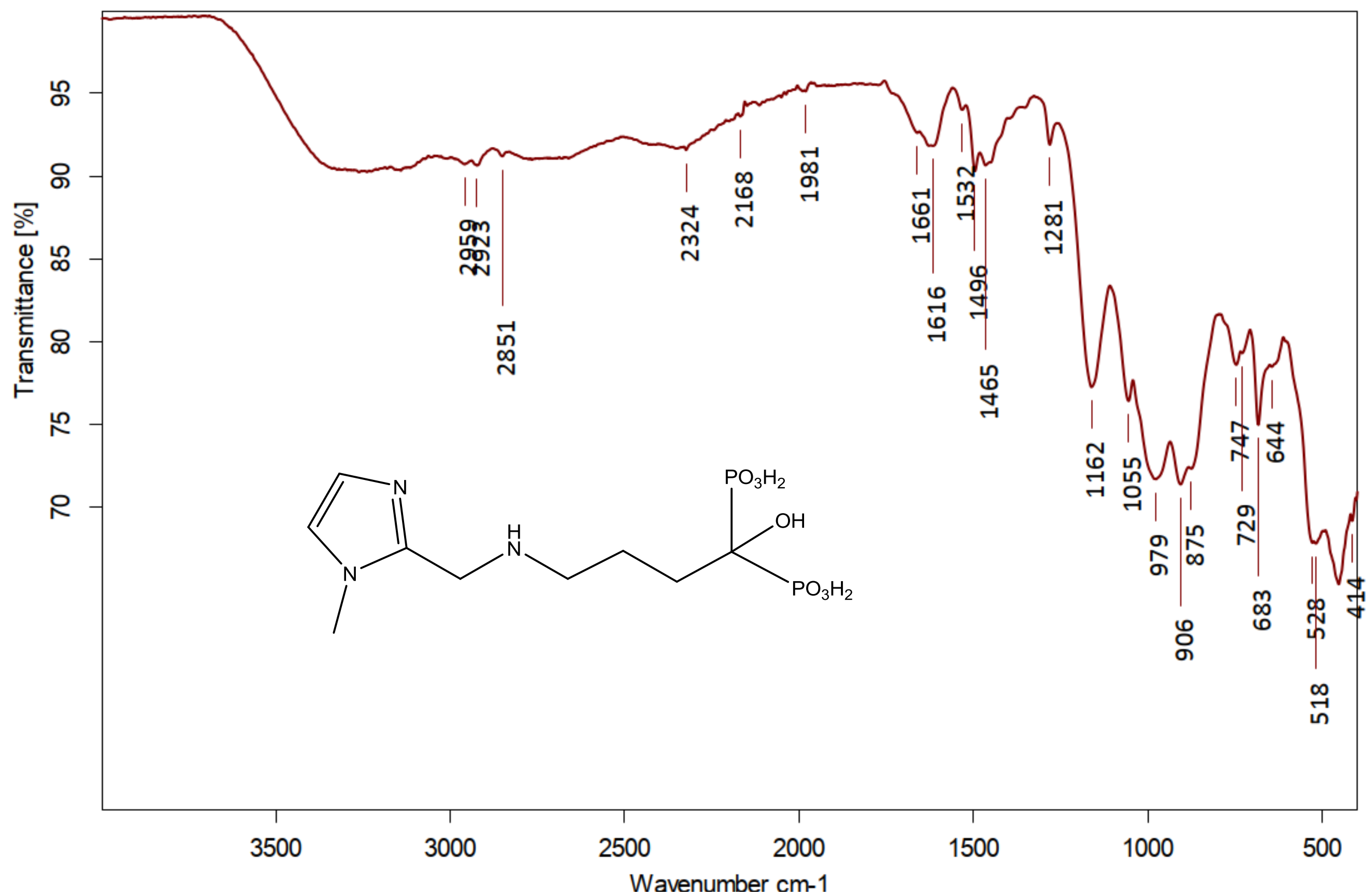

Figure S39. ATR-FTIR spectrum of compound **4g**.

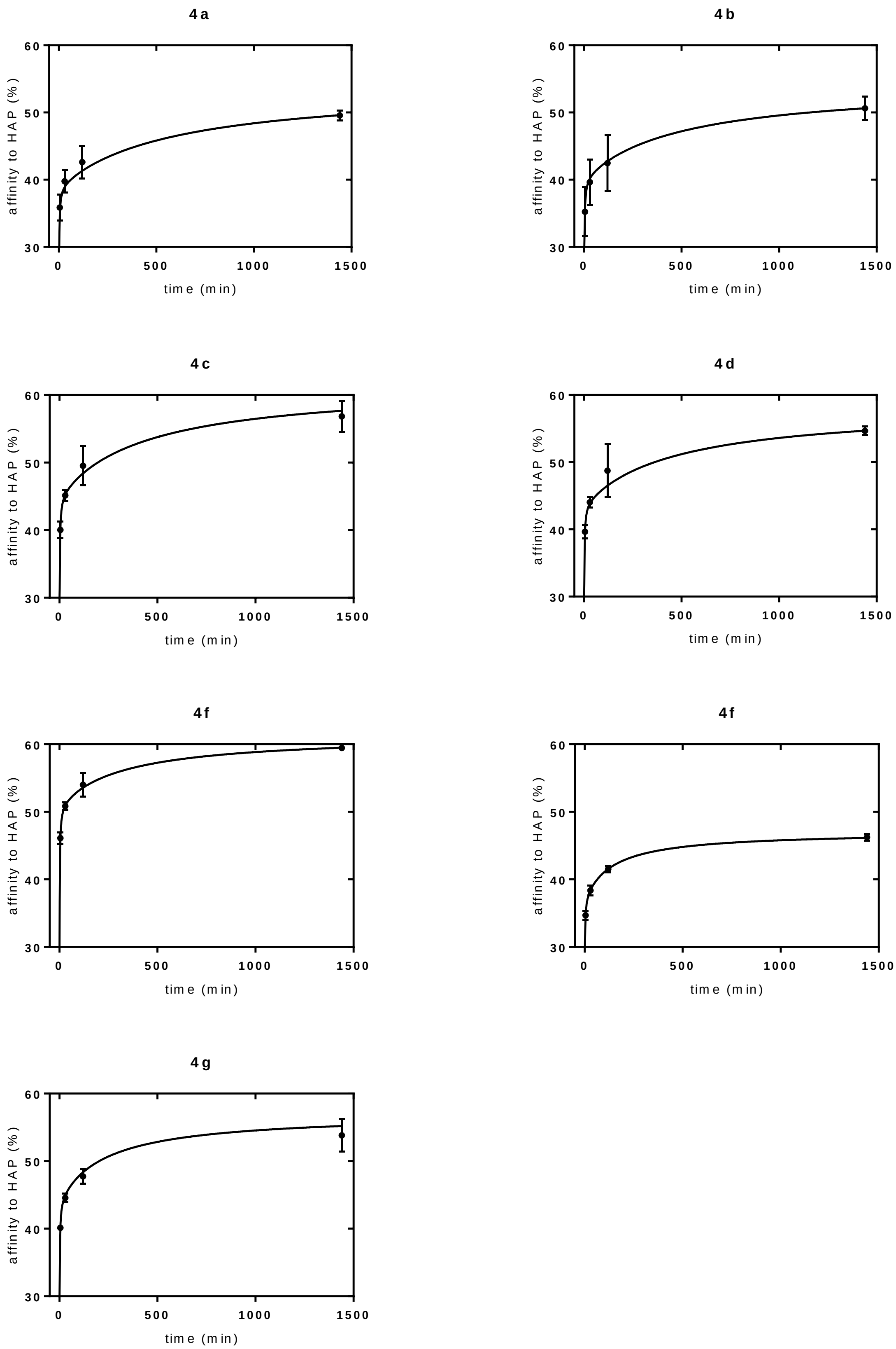

Figure S40. Time-dependent binding curves of the studied BPs to hydroxyapatite (HAP).

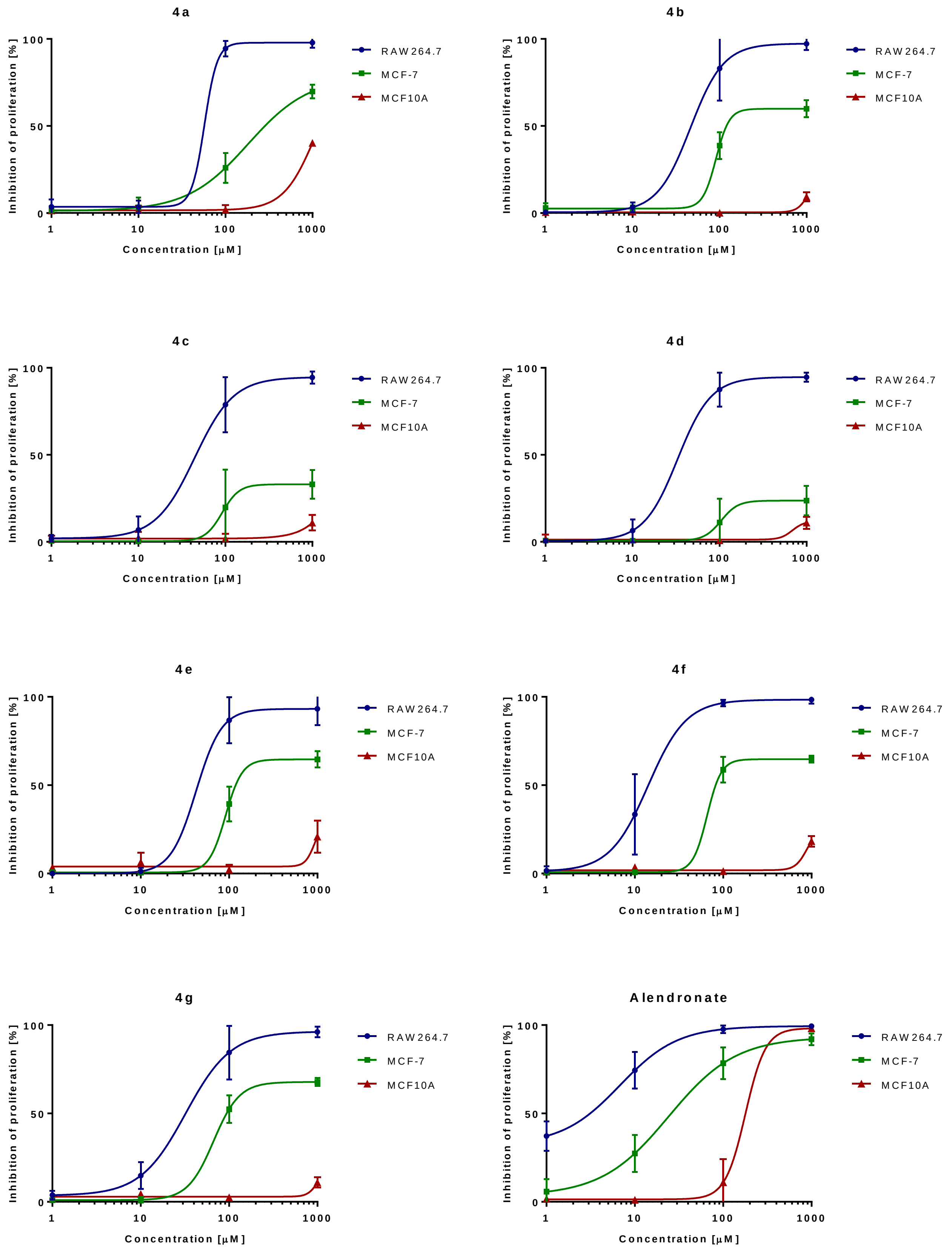

Figure S41. Dose–response curves for all BPs.
